# The genetic diversity of narcissus viruses related to turnip mosaic virus blur arbitrary boundaries used to discriminate potyvirus species

**DOI:** 10.1371/journal.pone.0190511

**Published:** 2018-01-04

**Authors:** Kazusato Ohshima, Shinichiro Mitoma, Adrian J. Gibbs

**Affiliations:** 1 Laboratory of Plant Virology, Department of Applied Biological Sciences, Faculty of Agriculture, Saga University, Saga, Japan; 2 The United Graduate School of Agricultural Sciences, Kagoshima University, Kagoshima, Japan; 3 Emeritus Faculty, Australian National University, Canberra, Australia; Oklahoma State University, UNITED STATES

## Abstract

*Narcissus* plants (*Narcissus tazetta* var. chinensis) showing mosaic or striping leaves were collected from around Japan, and tested for virus infections using potyvirus-specific primers. Many were found to be infected with a macluravirus and mixtures of different potyviruses, one third of them narcissus yellow stripe virus (NYSV)-like viruses. Genomes of nine of the NYSV-like viruses were sequenced and, together with four already published, provided data for phylogenetic and pairwise identity analyses of their place in the turnip mosaic virus (TuMV) phylogenetic group. Using existing ICTV criteria for defining potyvirus species, the narcissus viruses in TuMV group were found to be from five species; the previously described NLSYV, and four new species we call narcissus virus 1 (NV-1) and narcissus yellow stripe-1 to -3 (NYSV-1, NYSV-2 and NYSV-3). However, as all are from a single host species, and natural recombinants with NV-1 and NYSV-3 'parents have been found in China and India, we also conclude that they could be considered to be members of a single mega-species, narcissus virus; the criteria for defining such a potyvirus species would then be that their polyprotein sequences have greater than 69% identical nucleotides and greater than 75% identical amino acids.

## Introduction

Studies of the genetic diversity and evolutionary relationships of plant viruses are important for understanding plant virus epidemiology and for planning control measures [1–4]. Such studies have been done on cucumber mosaic virus [5]; rice yellow mottle virus [6]; geminiviruses [7–9], pararetroviruses [10], turnip mosaic virus (TuMV) and its relatives [11–16].

*Turnip mosaic virus* is a species in the family *Potyviridae*. All potyviruses are transmitted non-persistently by migrating aphids of many species [17]. Some potyviruses are also seed-borne. They have flexuous filamentous particles 700–750 nm long, each of which contains a single copy of their genome. Their genomes are single-stranded, positive-sense RNA molecules approximately 10,000 nucleotides long with one major open reading frame (ORF) that is translated into one large polyprotein and with a small overlapping ORF [18].

TuMV is a member of a clearly defined lineage of potyviruses, the TuMV phylogenetic group, all of which, except TuMV, have been isolated from monocotyledonous plants [3]. The TuMV group consists of Japanese yam mosaic virus (JYMV) [19–21], narcissus late season yellows virus (NLSYV) [22, 23], narcissus yellow stripe virus (NYSV) [23–27], scallion mosaic virus (ScaMV) [14, 28], wild onion symptomless virus (WoSV) [13] and TuMV [11, 12, 29].

Here we report a further study of TuMV group viruses from *Narcissus tazetta* var. chinensis plants collected in Japan. *Narcissus* is a genus of around 80 perennial monocotyledonous species with a centre of diversity around the Mediterranean, especially the Iberian Peninsula, but its species are also distributed across Europe and Asia, and *N*. *tazetta* was probably introduced into China and Japan from the Middle East along trading routes in prehistoric times [30]. *N*. *tazetta*, a feral, is known to be infected with several different potyviruses including NYSV, which was the first narcissus virus to be described [24–26, 31], and also NLSYV, Indian narcissus virus, lily mottle virus, narcissus degeneration virus (NDV), ornithogalum mosaic virus, cyrtanthus elatus virus A (CyEVA) and iris severe mosaic virus [15, 22, 27, 32–35]. Early attempts to resolve this complex proved to be difficult [36] before enzyme-linked immunosorbent assay (ELISA) tests were used. Most earlier studies [24–26] of these viruses investigated their biological characteristics, here we report a molecular phylogenetic study.

Our interest in narcissus viruses was aroused as *Narcissus* overwinters as perennial geophytic bulbs, and also occasionally disperses as seeds. These ecological features contrast with those of the annual crop and weed dicotyledonous hosts of TuMV and therefore, we reasoned, the phylogeny of potyviruses in *Narcissus* may contrast interestingly with that of TuMV in annual brassicas. Individual *Narcissus* plants are often infected with more than one virus [32], and potyviruses are not the only agents causing mosaic and striping diseases of them. The narcissus viruses have narrow host ranges, and NLSYV, NYSV and NDV are mostly limited to *Narcissus* in nature. For this project, we collected samples from plants of *N*. *tazetta* var. chinensis showing yellow mosaic or striped leaves around Japan. These were tested for potyvirus infections, single and multiple, including viruses of the TuMV phylogenetic group, and the complete genomic sequences of several narcissus virus isolates from Australia were determined recently [23], and their relationships found to be complex and unresolved. Thus, the relationship of selected isolates was determined using complete genomic sequences. In this study, the full length genomes, especially of NYSV-like viruses, were compared in an attempt to resolve, and better define, species boundaries in the TuMV phylogenetic group.

## Material and methods

### *Narcissus* plants

Wild and domestic *Narcissus* plants (*Narcissus tazetta* var. chinensis) showing mosaic and striped leaves were collected from different sites on the banks of rivers and fields, including home gardens and flower beds, in Japan during the winter and spring seasons of 2009–2015. We asked the owners for permission to collect samples from their properties. None of the samples were from ‘endangered’ species. Details of the plants, their places of origin, site types and years of isolation are shown in S1 Table.

### Detection, cloning and sequencing of narcissus virus genomes

The viruses were directly identified from the collected *Narcissus* leaves by reverse transcription and polymerase chain reaction (RT-PCR) using potyvirus-specific primer pairs which were expected to amplify all potyviruses, and by the partial sequencing of the cloned RT-PCR products as described below. The total RNAs were extracted using Isogen RNA extraction reagent (Nippon Gene, Tokyo, Japan) from the leaves of *Narcissus* plants (S1 Table). The RNAs were reverse-transcribed, and the cDNAs were amplified using high-fidelity PrimeSTAR GXL DNA Polymerase in PrimeScript II High Fidelity One Step RT-PCR Kit (TaKaRa Bio, Shiga, Japan). The RT-PCR conditions were: 45°C for 10 min for RT, one cycle of 94°C for 2 min, and 40 cycles of 98°C for 10 s, 45°C for 15 s and 68°C for 35 s. RT-PCR products of approximately 2,100 bp were amplified from *Narcissus* leaves using potyvirus-specific primer pairs [14, 15] modified from the previously reported primers [14, 37, 38], POTYNIBNOT4P (5’- GGGGCGGCCGCATATGGGGTGAGAGAGGTNTGYGTNGAYGAYTTYAAYAA -3’) and TU3T9M (5’- GGGGCGGCCGCT_15_−3’) for virus genome amplification (S1 Fig, S2 Table). The cDNAs were separated by electrophoresis in agarose gels and purified using a QIAquick Gel Extraction kit (Qiagen K.K., Tokyo, Japan). The RT-PCR products were cloned into NotI site of plasmid pZErO-2. We obtained three to fifteen independent clones from each virus-infected *Narcissus* plant. The nucleotide sequences (approximately 600–700 nucleotides) of parts of the amplified fragments (approximately 2,100 nucleotides long) of all clones were first determined using POTYNIB5P primer (5’- CGCATATGGGGTGAGAGAGG- 3’), a part of POTYNIBNOT4P (underlined) using a BigDye Terminator v3.1 Cycle Sequencing Ready Reaction kit (Applied Biosystems, Foster City, CA, USA) and an Applied Biosystems Genetic Analyzer DNA model 3130. Because large numbers of clones (1048 in total) were obtained (S1 Table), we first wished to identify the viruses in each *Narcissus* plant using 600–700 nucleotide sequences obtained by POTYNIB5P primer. Then the sequences of chosen clones of the remaining nuclear inclusion b (NIb) coding region, the complete coat protein (CP) coding region and the 3’ non-coding region (NIb-3’ region) were also determined in both directions using NYSV-like virus specific primers by primer walking. BLAST searches showed the cloned CP sequences to be closely related to one or other of the sequences of CyEVA, NDV, NLSYV and NYSV, or narcissus latent macluravirus (NLV) [15]. Note that the term “NYSV-like virus” refers to the narcissus viruses in the TuMV phylogenetic group, whose genomes can be amplified by POTYNIBNOT4P and TU3T9M primer pairs, but does not include NLSYV.

Similarly, three or four fragments covering the full genomic regions of NYSV-like viruses, including their NIb-3’ region, were amplified by RT-PCR (S1 Fig). For NY-CB5 and NY-EH173 isolates, two fragments, from the 5’ end to the 6Kda 1 protein (6K1) coding region (5’-6K1 region) and from the protein 3 (P3) to NIb coding regions (P3-NIb region), were amplified by RT-PCR using appropriate primers designed from sequences obtained in the present study and from those in the public nucleotide sequence databases (S2 Table). For NY-KM1P isolate, three fragments, from the 5’ end to the P3 (5’-P3 region), from the helper-component proteinase protein (HC-Pro) to nuclear inclusion a-proteinase (NIa-Pro) coding regions (HC-NIa region), and from the cylindrical inclusion protein (CI) coding region to CP coding regions (CI-CP region), were amplified by RT-PCR. For NY-HG16, NY-HG19, NY-HG27, NY-HR38, NY-KM1O and NY-OY1 isolates, three fragments, from the 5’ end to the CI coding region (5’-CI region), the HC-NIa and the CI-CP regions, were amplified by RT-PCR. Note that TU5T5P primer with NotI site (underlined) (5’-GGGGCGGCCGCAAAAAATATAAAAACTCAACACAACA-3’) is a degenerate primer of the 5’ end of TuMV phylogenetic group virus genomes. The RT-PCR products were cloned into the NotI site of plasmid pZErO-2. Two to four independent clones for each fragment were obtained. The overlapping regions of RT-PCR products were at least 300 nucleotides, and clones that had no mismatches in the regions were assembled to obtain full-genomic sequences. The nucleotide sequences of clones were determined in both directions by primer walking using more than fifty primers because the sequences of the isolates varied. Sequence data were assembled using BioEdit version 5.0.9 [39].

### Alignment of sequences

The alignment for Neighbour-net (NN), recombination and maximum likelihood (ML) phylogenetic analyses was made from the deduced amino acid sequences of the complete CP coding and polyprotein regions of NYSV-like viruses together with those of the same viruses from the public nucleotide sequence databases using outgroup sequences of NLSYV, JYMV, ScaMV, TuMV and WoSV. The alignments were made using CLUSTAL_X2 [40] with TRANSALIGN (kindly supplied by Georg Weiller) to maintain the alignment of the encoded amino acids, and the WEBprank server (http://www.ebi.ac.uk/goldman-srv/webprank/). The nucleotide sequences of degapped CP coding and polyprotein regions were 798 and 8940 nucleotides in length for each isolate. Finally, the NYSV-like virus sequences were also aligned without outgroup sequences, and directly checked for evidence of recombination (see below). Note that incomplete nucleotide sequences with ambiguous nucleotides in the public nucleotide sequence databases were not included in the analyses.

### Recombination analysis

Putative recombination sites were identified using the RDP [41], GENECONV [42], BOOTSCAN [43], MAXCHI [44], CHIMAERA [45] and SISCAN [46] programs as implemented in the RDP4 version 4.71 package [47]. These analyses were done using the default settings for the different detection programs and a Bonferroni corrected *P*-value cut-off of 0.05 or 0.01, and then isolates were identified as likely recombinants detected by three or more different methods with an associated *P*-value of <1.0×10^−6^. These analyses also assessed which non-recombinant sequences had regions that were closest to those from the recombinant sequences, indicating the likely lineages that provided those regions of the recombinant genomes. For convenience, we refer to them as the ‘parental isolates’ of the recombinants.

### Phylogenetic analysis

The phylogenetic relationships of the aligned sequences of complete CP coding and polyprotein regions of NYSV-like viruses were inferred using the NN method in SPLITSTREE version 4.11.3 [48] and ML tree in PhyML version 3.1 [49]. The best-fit model of nucleotide substitutions for each dataset was determined using jModelTest version 2.1.7 [50]; the general time-reversible substitution model with invariant sites and a gamma distribution and a proportion of invariable sites (GTR+I+T_4_) provided the best-fit for the TuMV phylogenetic group sequences. This model was selected in R [51] using the Bayesian information criterion, which has been shown to perform well with different datasets [52]. Branch support was evaluated by bootstrap analysis based on 1000 pseudoreplicates. The inferred trees were displayed by TreeView [53]. Neighbor-joining and minimum evolution trees were also constructed using MEGA7 [54].

### Nucleotide identity

The nucleotide sequence identities were estimated using EMBOSS Needle [55]. The pairwise nucleotide sequence identity scores were represented as a distribution plot using SDT version 1.2 [56]. The similarities were estimated using SIMPLOT version 3.5.1 with a window size of 200 nucleotides, and with the novel narcissus virus genomic sequences as queries. The similarities between the genome sequences of novel narcissus virus isolates and the viruses in TuMV phylogenetic group [3, 14, 15] were analysed.

## Results and discussion

### 3’ terminal region

#### Detection and identification of viruses in the *Narcissus* samples

One hundred and eighty-eight symptomatic *Narcissus* plants were collected during the 2009–2015 growing seasons in Japan (S1 Table), and 119 plants (63.3%) were found to be infected with at least one potyvirus (Table 1). A total of 1048 clones of the NIb protein coding regions of all of the clones were analysed using POTYNIB5P primer (680,000 nucleotides in total) and identified by sequence comparisons. One hundred nineteen out of 188 collected plants (63.3%) were found to be infected with one or more viruses in *Potyviridae* family (NYSV, NLSYV, CyEVA, NDV, OrMV and NLV) and 42 plants (22.3%) were coinfected with at least one potyvirus and one macluravirus; (Table 1, S1 Table). Fifty seven plants (30.3%) were infected with NYSV-like viruses.

**Table 1:** Detection of viruses in the family *Potyviridae* and narcissus yellow stripe virus (NYSV)-like viruses in Japan.

	Numbers of plants		
	Examined	detected	
District		Virus in the family *Potyviridae*^a^ (%)	NYSV-like virus (%)
Hokkaido	24	9 (37.5)	0 (0.0)
Tohoku	25	7 (28.0)	4 (16.0)
Kanto	30	23 (76.7)	10 (33.3)
Chubu	19	15 (78.9)	4 (21.1)
Kinki	20	12 (60.0)	8 (40.0)
Chugoku	9	8 (88.9)	4 (44.4)
Shikoku	15	10 (66.7)	5 (33.3)
Kyushu and Okinawa	46	35 (76.1)	22 (47.8)
Total	188	119 (63.3)	57 (0.3)

^a^ This includes narcissus late season yellows virus (NLSYV), cyrtanthus elatus virus A (CyEVA), narcissus latent virus (NLV), narcissus degeneration virus (NDV) and/or ornithogalum mosaic virus (OrMV).

**Fig 1:**
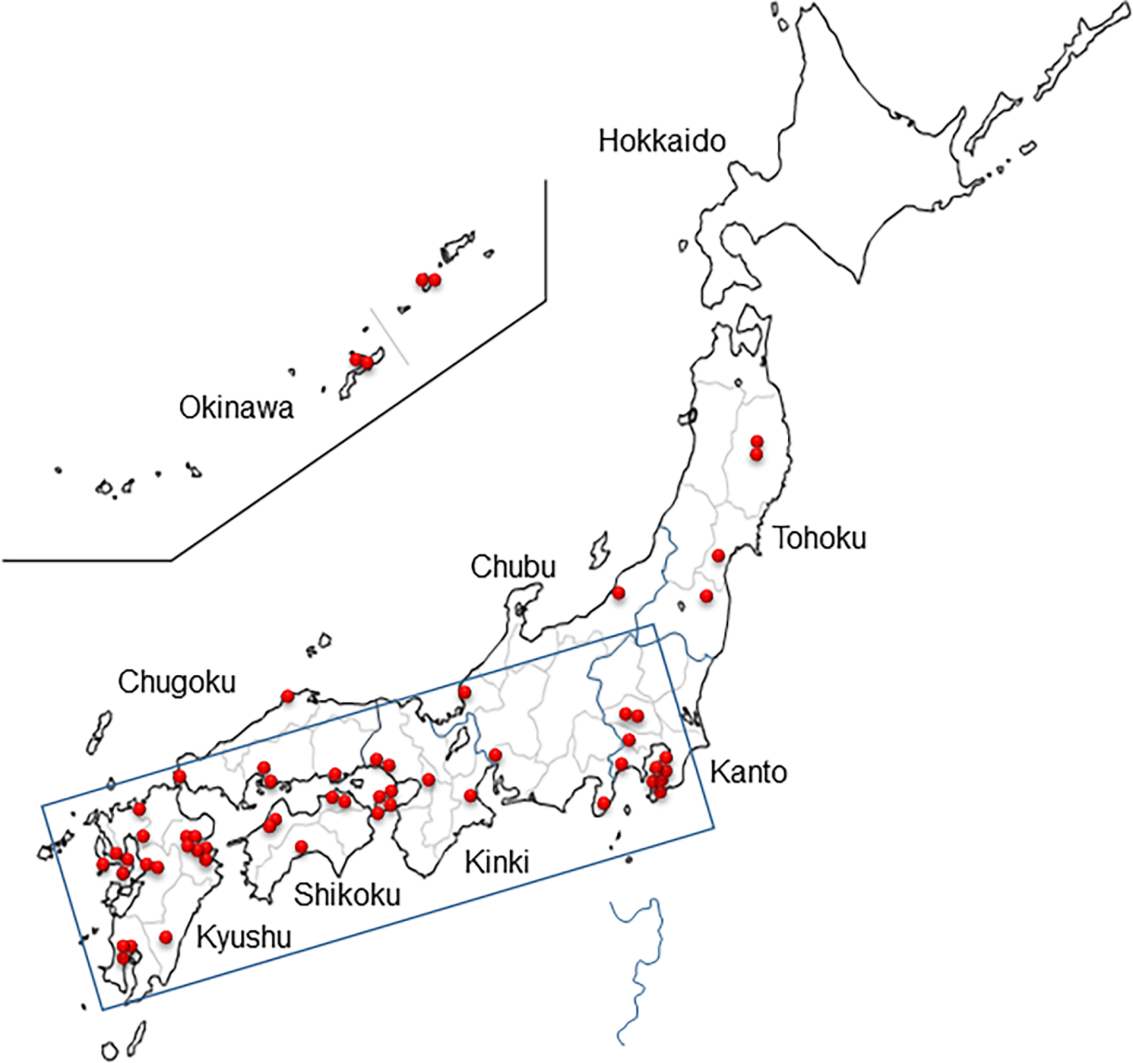
Location of *Narcissus* plants infected with narcissus yellow stripe virus (NYSV)-like viruses. Red dots show the narcissus plants infected with NYSV-like viruses. The major area of *Narcissus* plants infected with NYSV-like viruses is boxed. The sampling sites of *Narcissus* plants in Japan are listed in S1 Table.

We chose 91 clones of the NYSV-like viruses using the sequences obtained by the POTYNIB5P primer, and then analysed the remaining region of their NIb-3’ sequences (S1 Table). The nucleotide sequences of CP regions of NYSV-like viruses determined in this study were 819–822 nucleotides in length. NYSV-like virus infections were mostly found in the *Narcissus* plants collected from the west of Japan (Fig 1). This paper reports studies of these NYSV-like isolates; we will describe the results of work on the other viruses elsewhere.

#### Phylogenetic analysis of coat protein genes of NYSV-like viruses

**Fig 2:**
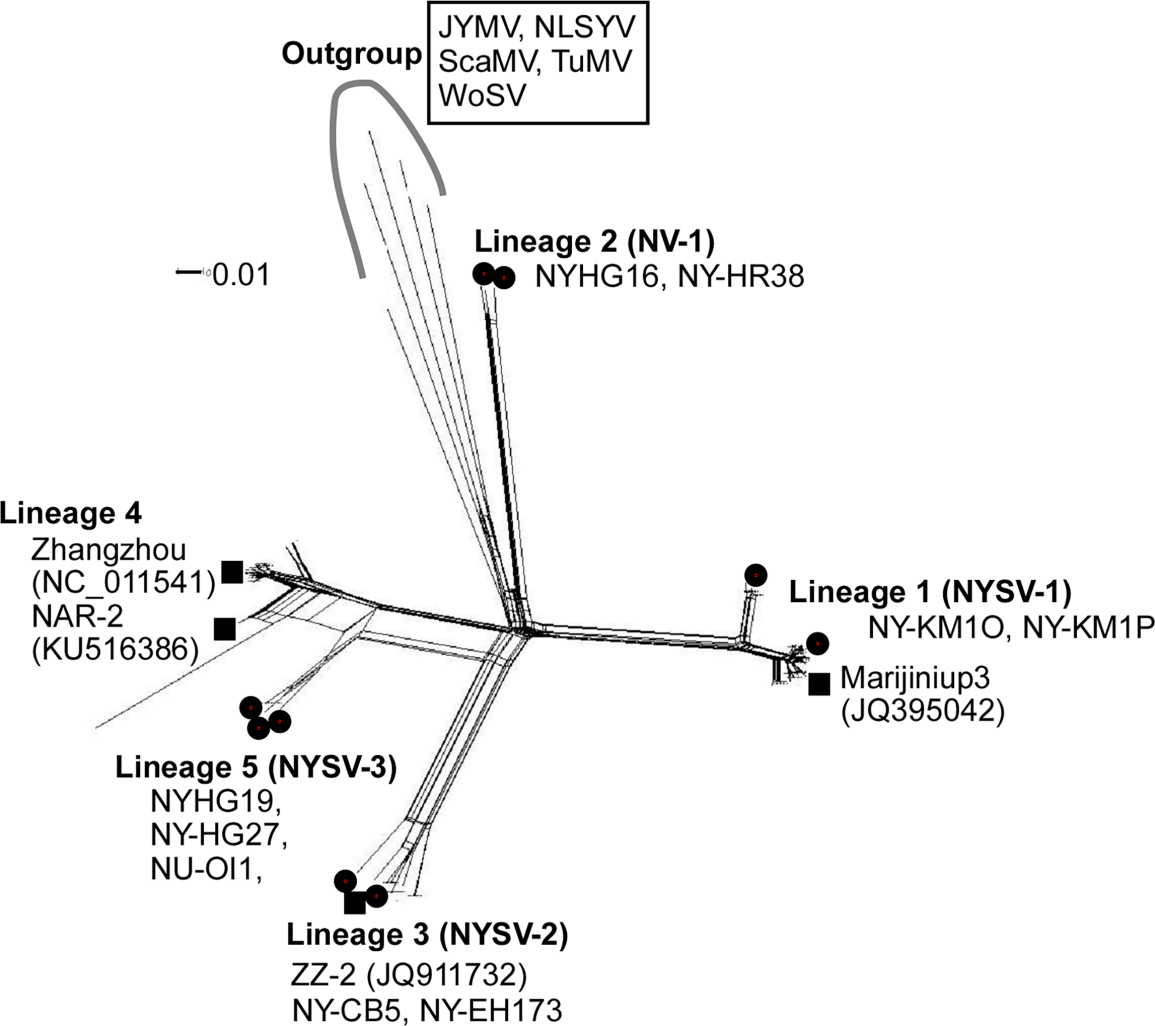
Phylogenetic networks of turnip mosaic potyvirus phylogenetic group viruses. The sequences of complete coat protein coding region of narcissus yellow stripe virus (NYSV)-like viruses obtained in this study with those of outgroup sequences of Japanese yam mosaic virus (JYMV), narcissus late season yellows virus (NLSYV), scallion mosaic virus (ScaMV), turnip mosaic virus (TuMV) and wild onion symptomless virus (WoSV). Isolates used in this study and isolates with accession numbers obtained from the public nucleotide sequence databases were listed. Black dots indicate the isolates used for full genomic sequencing. For details of lineages, see text and Fig 3.

The 91 CP sequences determined in this study, together with 46 sequences obtained from the public nucleotide sequence databases, were analysed. The length of aligned CP sequences was 798 nucleotides after gaps were removed. SPLITSTREE analyses using both nucleotide and amino acid sequences showed similar topology and found a reticulated phylogenetic network linking the CP genes. Fig 2 shows the NN tree constructed using nucleotide sequences. However, when the sequences were assessed for evidence of recombination, none was found, but there were five major lineages in the NN tree. Nine isolates were selected for full genomic sequencing to obtain at least one full genomic sequence in each lineage, and to assess their relationships with the four previously reported isolates from Australia and China. We chose NY-KM1O and NY-KM1P isolates from lineage 1, NY-HG16 and NY-HR38 isolates from lineage 2, NY-CB5 and NY-EH173 from lineage 3, and NY-HG19, NY-HG27 and NY-OI1 isolates from lineage 5 for full genomic sequencing.

### Relationships of the NYSV-like viruses

#### Genomic sequences of selected NYSV-like viruses

We compared the complete genomes of the nine NYSV-like viruses that we sequenced together with seven NYSV and NLSYV sequences from the public nucleotide sequence databases (JQ326210, JQ395042, JQ911732, JX156421, KU516386, NC_011541, NC_023628). The genomes of Japanese isolates of NYSV-like viruses were 9626–9639 nucleotides in length excluding the 5’ end 24 nucleotide primer sequences (S2 Table). The regions encoding the protein 1 (P1), HC-Pro, P3, overlapping ORF, 6K1, CI, 6Kda 2 protein (6K2), genome-linked viral protein (VPg), NIa-Pro, NIb and CP proteins were 951–963, 1374, 1062, 181–261, 156, 1932, 159, 570–573, 729, 1551 and 822 nucleotides long respectively, and, for most analyses, the 5’ end primer sequences were omitted leaving only the region encoding the polyprotein. The lengths of 5’ non-coding region, P1, VPg and 3’ non-coding region varied among narcissus viruses in TuMV phylogenetic group. All of the motifs reported for different potyvirus encoded proteins were found. The cleavage sites of protein coding regions were similar for all narcissus viruses, except those between the P1 and HC-Pro coding regions, and between the P3 and 6K1 coding regions (S3 Table). The new genomic sequences determined in this study are available in the public nucleotide sequence databases with Accession numbers LC314391-LC314399.

#### Recombinant analysis

SPLITSTREE was used to analyse the polyprotein-encoding regions of the 13 NYSV-like viruses together with NLSYV and outgroup sequences (S2A Fig). The length of the aligned polyprotein gene sequences was 8940 nucleotides. The analysis produced a reticulated phylogenetic network. The reticulation indicated the presence of recombinant sequences, so the sequences were analysed for evidence of recombination using the RDP4 suite of programs.

One unequivocal recombination site was found in the middle of the CI coding region (near nt 4600) of the NYSV Zhangzhou sequence (NC_011541) as previously reported [27]. The genomes most closely related to the two regions of the NYSV Zhangzhou isolate are those of NY-HR38 and NY-OI1 isolates with statistical support (i.e. *p*-values 1.3×10^−301^–9.4×10^−7^) in the all six programs in the RDP4 software. The NYSV NAR-2 isolate from India was also found to be a multiple recombinant with parental isolates resembling NY-HR38/NY-HG16 (major) and NY-HG19 (minor) or NY-OI1 (major) and NY-HG19 (minor) isolates, and all sites were supported by clear *p*-values (6.7×10^−145^–8.93×10^−17^) in the all six programs. The graph obtained by the original SiScan software is shown and the site was also supported by clear Z-values (>1×10^3^) for both 5’ and 3’ parents (S3 Fig). When the NYSV Zhangzhou and NAR-2 sequences were removed, the reticulate nodes disappeared from the NN tree of the remainder (S2B Fig), and so these two recombinant sequences were omitted from most of the phylogenetic analyses.

#### Pairwise relationships and the ICTV criteria

The International Committee on Taxonomy of Viruses (ICTV) discriminates members of different potyvirus species using pairwise sequence comparisons. The polyprotein ORFs of isolates of different species in a large sample of potyviruses were found to have less than 76% pairwise nucleotide identity and less than 82% pairwise amino acid identity [57, 58]. Therefore, a preliminary analysis of the polyprotein genes of all the TuMV group viruses, including representative sequences of JYMV, NLSYV, ScaMV, TuMV and WoSV was done using the SDT method, which showed that the non-recombinant NYSV-like viruses probably formed five species. These groupings were confirmd by an EMBOSS Needle analysis. Table 2 lists the identities between each of the narcissus virus-1 (NV-1, see the section ‘Phylogenetic relationships’) sequences and those of other members of the group. It can be seen that all are from separate species when judged by either their nucleotide or amino acid sequence identities. When however all pairwise identities of the NYSV-like viruses are calculated (Table 3), some are found to be separate species by their nucleotide identities, but not by their amino acid identities. We also calculated the nucleotide identities of the P1 and cytoplasmic inclusion protein (CI) coding region (S4 Table) as this includes the most variable parts of the polyprotein genes, and again found that the sequences of the narcissus viruses did not fall neatly into five taxa as judged by the ICTV criteria.

**Table 2:** Comparison of the percentage identical nucleotides and amino acids in the polyprotein region between the basal narcissus virus 1 (NV-1) isolates^a^ and other turnip mosaic virus phylogenetic group virus isolates.

	NY-HG16 (%)	NY-HR38 (%)
wild onion symptomless virus (WoMV)		
TUR256-1 (NC_030391)	64.3^b^ (68.1^c^)	64.7 (68.2)
TUR256-2 (LC159495)	64.3 (68.1)	64.7 (68.2)
scallion mosaic virus (ScaMV)		
China (NC_003399)	62.4 (62.1)	62.3 (62.1)
turnip mosaic virus (TuMV)		
OM (AB701690)	61.6 (61.0)	61.8 (60.9)
ASP (AB701697)	62.1 (61.5)	61.8 (61.3)
Japanese yam mosaic virus (JYMV)		
China (KJ701427)	61.7 (59.8)	62.6 (59.9)
JY1 (AB016500)	61.7 (59.3)	61.7 (59.2)
narcissus late season yellows virus (NLSYV)		
Marijiniup8 (NC_023628)	69.9 (75.8)	69.6 (75.7)
Marijiniup9 (JX156421)	69.7 (76.0)	69.4 (75.9)
Zhangzhou (JQ326210)	70.1 (76.0)	70.3 (76.0)
narcissus yellow stripe virus-like virus (NYSV-like virus)		
Marijiniup3 (JQ395042)	70.4 (77.2)	70.5 (77.5)
NY-CB5	69.4 (76.7)	69.2 (76.5)
NY-EH173	69.1 (76.7)	69.1 (76.4)
NY-HG19	70.4 (77.4)	70.5 (77.4)
NY-HG27	70.4 (77.6)	70.9 (77.3)
NY-KM1O	70.6 (77.0)	70.6 (77.4)
NY-KM1P	70.2 (77.4)	70.1 (77.6)
NY-OI1	70.9 (77.6)	70.7 (77.6)
ZZ-2 (JQ911732)	69.8 (76.7)	69.6 (76.6)

^a^ Narcissus virus 1 isolates were basal isolates of narcissus viruses in turnip mosaic virus phylogenetic group (see Fig 3).

^b^ Nucleotide identity. The identities were calculated using EMBOSS Needle [55].

^c^ Amino acid identity

**Table 3:** Comparison of the percentage identical nucleotides (top right) and amino acids (bottom left) in the polyprotein region of narcissus yellow stripe virus-like virus and narcissus late season yellows virus isolates.

Virus and isolate	narcissus virus 1		narcissus late season yellows virus			narcissus yellow stripe virus-like virus								
	NV-1					NYSV-1			NYSV-2			NYSV-3		
	NY-HG16	NY-HR38	Marijiniup8	Marijiniup9	Zhangzhou	Marijiniup3	NY-KM1O	NY-KM1P	NY-CB5	NY-EH173	ZZ-2	NY-HG19	NY-HG27	NY-OI1
NY-HG16	-	***95*.*7***	69.9	69.7	70.1	70.4	70.6	70.2	69.4	69.4	69.8	70.4	70.4	70.9
NY-HR38	***97*.*4***	-	69.6	69.4	70.3	70.5	70.6	70.1	69.2	69.1	69.6	70.5	70.9	70.7
Marijiniup8	75.8	75.7	-	***82*.*3***	***82*.*2***	70.7	70.5	70.8	70.2	70.2	69.7	70.6	70.7	71.0
Marijiniup9	76.0	75.9	***91*.*7***	-	***90*.*0***	71.0	70.6	71.0	70.3	70.4	70.1	70.9	71.2	70.8
Zhangzhou	76.0	76.0	***91*.*7***	***95*.*8***	-	71.3	71.5	71.6	71.5	71.2	71.7	75.6	75.8	71.7
Marijiniup3	77.2	77.5	79.5	79.3	79.5	-	***96*.*3***	***91*.*7***	72.3	72.5	72.3	72.5	72.6	72.8
NY-KM1O	77.0	77.4	79.3	78.9	79.3	***97*.*7***	-	***91*.*9***	72.4	72.3	72.5	72.7	72.6	73.4
NY-KM1P	77.4	77.6	79.3	78.9	79.3	***96*.*3***	***96*.*4***	-	72.8	72.5	72.5	72.4	72.6	73.6
NY-CB5	76.7	76.5	76.9	77.4	77.2	81.3	81.4	81.1	-	***95*.*2***	***93*.*6***	73.9	74.1	73.1
NY-EH173	76.7	76.4	76.9	77.5	77.2	81.3	81.3	81.0	***98*.*0***	-	***93*.*9***	73.7	73.5	73.3
ZZ-2	76.7	76.6	77.0	77.5	77.2	81.2	81.2	80.8	***97*.*0***	***97*.*0***	-	73.7	73.7	73.9
NY-HG19	77.4	77.4	78.0	78.4	78.4	***82*.*1***	***82*.*3***	***82*.*1***	***83*.*0***	***82*.*9***	***83*.*3***	-	***96*.*0***	***81*.*9***
NY-HG27	77.6	77.3	78.1	78.5	78.5	***82*.*2***	***82*.*4***	***82*.*2***	***83*.*2***	***83*.*0***	***83*.*3***	***97*.*9***	-	***81*.*7***
NY-OI1	77.6	70.7	78.4	78.6	78.9	***82*.*7***	***82*.*6***	***82*.*4***	***82*.*8***	***83*.*2***	***83*.*2***	***91*.*8***	***91*.*8***	-

Percent identities of the polyprotein coding regions were calculated by EMBOSS Needle [59]. The identity values greater than the thresholds of species demarcation criteria are shown in bold and italicized letter. Potyvirus species demarcation criteria of polyprotein coding regions are <76% nucleotide identity and <82% amino acid identity [57, 58].

The similarities and differences we observed were uniformly distributed through the sequences as was shown by the SIMPLOT method. S4A Fig shows, for example, comparisons using a NV-1 sequence (NY-HG16) as the query sequence. The graph shows that the most parts of NV-1 polyprotein sequences are less than 76% similar to those of JYMV, ScaMV, TuMV-OM, NLSYV and WoSV, but not that of the other NV-1 sequence (NY-HR38). Furthermore, comparisons between a NYSV-1 sequence (NY-KM1P) and those of NYSV-like viruses were shown (S4B Fig).

#### Phylogenetic relationships

**Fig 3:**
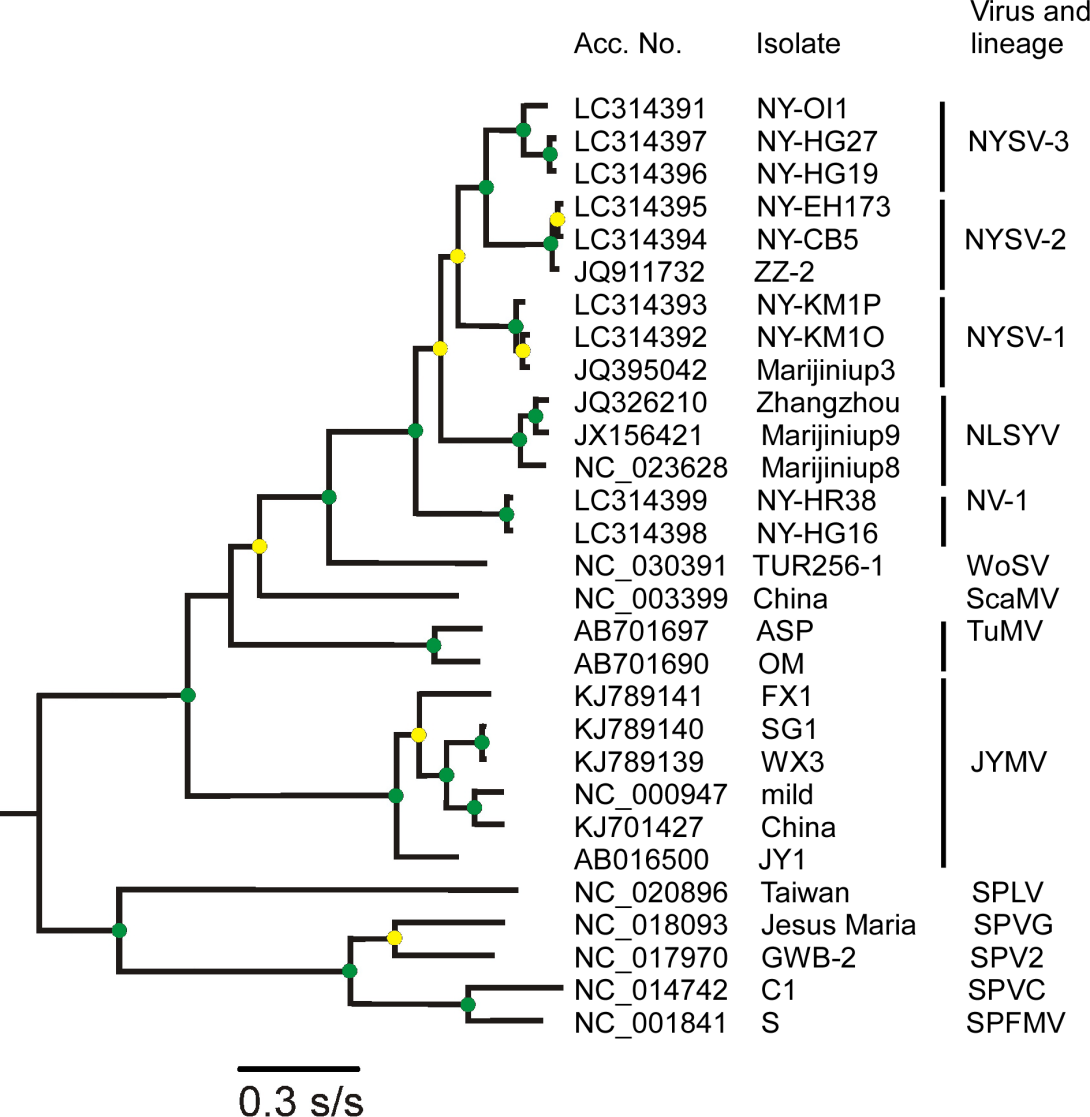
Maximum-likelihood tree showing the relationships of the polyprotein sequences of 14 non-recombinant narcissus viruses. The amino acid sequences of polyprotein (major open reading frame) regions of Japanese yam mosaic virus (JYMV), scallion mosaic virus (ScaMV), turnip mosaic virus (TuMV) and wild onion symptomless virus (WoSV) related to TuMV phylogenetic group were used. Sweet potato latent virus (SPLV), sweet potato virus 2 (SPV2), sweet potato virus C (SPVC), sweet potato virus G (SPVG) and sweet potato feathery mottle virus (SPFMV) were used as outgroup sequences. Circles at each node indicate the statistical support using the SH method, green circle; SH 1.0 (full support), yellow circle; 0.8 < SH <1.0. (between SH 0.8 and SH 0.9999). Horizontal branch lengths are drawn to scale with the bar indicating 0.1 nt replacements per site. The calculated tree was displayed by TREEVIEW [53].

The relationships among the narcissus potyviruses and their relatives was most clearly shown by phylogenetic analyses. Fig 3 shows the ML tree of the polyproteins of the non-recombinant NYSV-like viruses together with representative sequences of JYMV, NLSYV, ScaMV, TuMV, WoSV and the reference sequential series of five clusters which we call NV-1 (basal), NLSYV (as before), NYSV-1, NYSV-2 and NYSV-3 (distal). The NV-1 cluster consists of two new isolates from Japan, NY-HG16 and NY-HR38 (Accession numbers LC314398 and LC314399), NLSYV is of isolates previously reported from China and Australia, Zhangzhou and Marijiniup8 and 9 (JQ 326210, NC_023628, and JX156421), NYSV-1 includes the Mariginiup3 isolate of NYSV (JQ395042) and two new Japanese isolates (NY-KM10 and NY-KM1P; LC314392, LC314393), NYSV-2 is of the ZZ-2 Chinese isolate (JQ911732) and two new Japanese isolates, NY-CB5 and NY-EH173 (LC314394 and LC314395), and NYSV-3 is of three new Japanese isolates NY-NY-OI1, NY-HG19 and NY-HG27 (LC314391, LC314396 and LC314397).

Although these clusters were detected using a small number of genomic sequences, they are monophyletic and have strong statistical support in ML phylogenetic analyses. Their nucleotide sequences and the encoded amino acid sequences gave closely similar trees; Fig 3 shows the tree calculated from the encoded amino acid sequences as it had greater statistical support for the five nodes sequentially linking the clusters than in the nucleotide tree (mean SH support 0.952, rather than 0.80), furthermore the patristic distance graph comparing the nucleotide and amino acid trees (S5 Fig) showed their concordance. The same tree topologies were also obtained by neighbor-joining and minimum evolution methods implemented in MEGA7. The inclusion of another 18 Japanese NYSLV isolates did not affect the topology of the taxonomy, nor the statistical support for it (we will describe the results of NYSLV population elsewhere). This crown group of narcissus viruses is most closely related to WoSV [13], which was isolated from a symptomless wild onion collected in Iznik, Turkey.

#### Two consequent questions: one taxonomic, the other biological

Our phylogenetic analyses show that the narcissus viruses fall into five statistically robust clusters, but these do not completely coincide with the groupings indicated by the pairwise comparisons used by the ICTV, probably because pairwise sequence identities computed from multiple independent pairwise alignments are not the same as those computed from multiple sequence alignments (i.e. phylogenetic analyses), and are less informative [56]. However, the narcissus viruses pose a particular problem as they have all been isolated from *Narcissus*, and in *Narcissus* give variable symptoms which do not recur every year. Furthermore, no reliable experimental differential herbaceous host has been found for them [26, 36]. Our phylogenetic analyses, however, indicate that there are five clusters of narcissus viruses, and confirms that the broader taxonomic question [23] is whether we should consider them to be five species or one.

Cross-protection, which was independently discovered by several researchers [59–61], was widely used in the past as a criterion for distinguishing plant virus species, but no specific tests have been done to check whether the NYSV-like viruses including NLSYV cross-protect against one another, but they often occur in mixtures in nature.

Genetic recombination is perhaps also emerging as an alternative biologically-based test of relatedness. Intra-species recombination is common in most potyvirus populations [3, 12, 62, 63], however, as reported here, it seems to be uncommon among narcissus viruses compared with other potyviruses [12, 62, 64–66], although only a small number of genomic sequences of narcissus viruses were analysed in this study. By contrast, inter-species recombination in potyviruses has rarely been reported; watermelon mosaic virus (WMV) and soybean mosaic virus (SMV) in potyviruses [67] is possibly one example. Thus, our finding that the NYSV Zhangzhou isolate (NC_011541) from China and the NYSV NAR-2 isolate (KU516386) from India, are distinct recombinants between NV-1 and NSYV-3 'parents' is especially noteworthy. It indicates either that these are interspecies recombinants, or that NV 1 and NSYV-3 are members of a single species, the boundaries of which are wider than those set by the ICTV.

In the early 20th century, plant virus species were distinguished from one another by biological characters, especially host range and symptoms, and many groupings made that way were subsequently found to be of immunologically and genetically related viruses. However recently, the process has been reversed, first the genetics is studied and then the biology, and although this is the only option for viral gene sequences obtained by metagenomic studies [68], it need not be for viruses that have been isolated in the conventional way. So for viruses like the potyviruses of *Narcissus*, we can still ask whether virus taxonomists should place viruses into neat genetically defined boxes and then name them, or whether it is better, if possible, to aid human communication by first defining "collections of strains whose known properties are so similar that there seems little value in giving them separate names." [69]. If, as we believe, it is the latter, then the narcissus viruses in TuMV phylogenetic group would be considered a single species, and the genetics we report merely indicates that the ICTV criteria for potyvirus species should be revised so that the polyprotein sequences of a single potyvirus species may differ by up to 31%, not 24%, pairwise nucleotide sequence identity and/or 25%, not 18%, pairwise amino acid sequence identity. However, the ICTV species demarcation limits are suggestions only, and sequence identity is one of several criteria that can be used to distinguish potyvirus species, although pairwise sequence identity is the one most commonly used these days.

The biological question highlighted by our studies is to ask what evolutionary process has caused the component populations of narcissus viruses to form a series of five phylogenetically distinct clusters. It could be a result of chance divergences, or it could be the result of adaptation to, or co-evolution with, an ancient, diverse though small, host population [70, 71]. Another possible scenario that explains the apparent speciation of the basal narcissus viruses are that they have naturally diversified in some of the other approximately 80 narcissus species across their natural range. Several of these species have been domesticated and they are often bulked up together in bulb nurseries. Nurseries provide opportunities for viruses to transmit to new hosts species. However, only further studies of the genetics of the narcissus viruses and their hosts will reveal the answers.

## Supporting information

S1 Fig Location of amplified fragments by reverse transcription and polymerase chain reaction. Arrow indicates primers used to amplify cDNA. Isolates are shown in parenthesis. The primer sequences are listed in S2 Table.
(TIF)

S2 Fig Phylogenetic networks of turnip mosaic virus phylogenetic group viruses. The sequences of polyproteins of narcissus yellow stripe virus (NYSV)-like viruses obtained in this study with those of outgroup sequences of Japanese yam mosaic virus (JYMV), narcissus late season yellows virus (NLSYV), scallion mosaic virus (ScaMV), turnip mosaic virus (TuMV) and wild onion symptomless virus (WoSV). Isolates with accession numbers were obtained from the public nucleotide sequence databases. Isolates NYSV Zhangzhou (NC_011541) and NYSV NAR-2 (KU516386) were added (A) or removed (B), and the trees were constructed.
(TIF)

S3 Fig Graphs showing SISCAN version 2 analysis of the polyprotein nucleotide sequence of isolate NYSV-NAR-2 (KU516386) with that of NY-HR38 (black line) and that of NY-HG19 (gray line). The sequences of NY-HR38 and NY-HG19 isolates represent the likely parental sequences of NYSV NAR-2 isolate. Note the support (i.e. *z*-value >3.0) for NYSV NAR-2 isolate being more closely related to NY-HR38 than NY-HG19 isolates in total nucleotide site analysis (see Table 2). The nucleotide positions are shown relative to the end of the degapped genome using the numbering of NAR-2 isolate. Arrows indicate the recombination sites of isolate NAR-2 identified using NY-HR38 and NY-HG19 sequences. For the graph, each window comparison involved sub-sequences of 100 nucleotides with a 50 nucleotides step between window positions.
(TIF)

S4 Fig Similarity plot of polyprotein nucleotide sequences of narcissus viruses and the viruses in turnip mosaic phylogenetic group. Isolates NY-HG16 (A) and NY-KM1P (B) were used as the query isolate. The similarities were estimated using SIMPLOT version 3.5.1 with a window size of 200 nt.
(TIF)

S5 Fig Graph comparing the patristic distances in the nucleotide and amino acid trees. (TIF)

S1 Table Collection sites and the results of genetic diagnosis of *Narcissus* plants in this study. ^a^ Rows in brown show that *Narcissus* plants were infected with narcissus yellow stripe virus (NYSV)-like virus, whereas rows in blue show that those were infected with narcissus late season yellows virus (NLSYV), cyrtanthus elatus virus A (CyEVA), narcissus latent virus (NLV), narcissus degeneration virus (NDV) or ornithogalum mosaic virus (OrMV).
^b^ Number of clone sequenced for approximately 600–700 bp by POTYNIB5P primer. ^c^ Not detected. ^d^ Number of clone for NYSV-like virus sequence.
(PDF)

S2 Table Primers used to amplify RT-PCR products in this study. ^a^ Correspond to the genome of Chinese isolate [1]. ^b^ P1; Protein 1, HC-Pro; Helper component-proteinase protein, P3; Protein 3, 6K1; 6Kda 1 protein, CI; Cylindrical inclusion protein, 6K2; 6Kda 2 protein, VPg; Genome-linked viral protein, NIa-Pro; Nuclear inclusion a-proteinase protein, NIb; Nuclear inclusion b protein, CP; Coat protein. ^c^ R; G+A, Y; C+T, S; G+C, M; A+C, W; A+T, K, T+G, V; A+C+G, D; A+T+G, H; A+T+C, N; A+T+G+C, GCGGCCGC; NotI restriction site. ^d^ Underlined sequence is the position of POTYNIB5P primer (5’- CGCATATGGGGTGAGAGAGG- 3’) used for sequencing.
(PDF)

S3 Table Tentative amino acid residues at the cleavage sites between protein coding regions. ^a^ NYSV; narcissus yellow stripe virus, NLSYV; narcissus late season yellows virus. ^b^ P1; protein 1, HC-Pro; helper component-proteinase protein, P3; protein 3, 6K1; 6kda 1 protein, CI; cylindrical inclusion protein, 6K2; 6kda 2 protein, VPg; genome-linked viral protein, NIa-Pro; nuclear inclusion a-proteinase protein, NIb; nuclear inclusion b protein, CP; coat protein.
^c^ Numbers colored in red show different amino acid residues at cleavage sites.
(PDF)

S4 Table Comparison of the percentage identical nucleotides in the protein 1 (bottom left) and cylindrical inclusion protein (top right) coding regions of narcissus yellow stripe virus-like virus and narcissus late season yellows virus isolates. Percent identities of protein 1 and cytoplasmic inclusion coding regions were calculated by EMBOSS Needle [2]. The identity values greater than the thresholds of species demarcation criteria are shown in red letter. Potyvirus species demarcation criteria of protein 1 and cytoplasmic inclusion coding regions are <58% and <76% nucleotide identities, respectively [3, 4].
(PDF)
