# Supplementary figures and images for: The genetic diversity of narcissus viruses related to turnip mosaic virus blur arbitrary boundaries used to discriminate potyvirus species
Source: PLoS One. 2018 Jan 4;13(1):e0190511. doi: 10.1371/journal.pone.0190511 (PMC5754079; doi:10.1371/journal.pone.0190511)

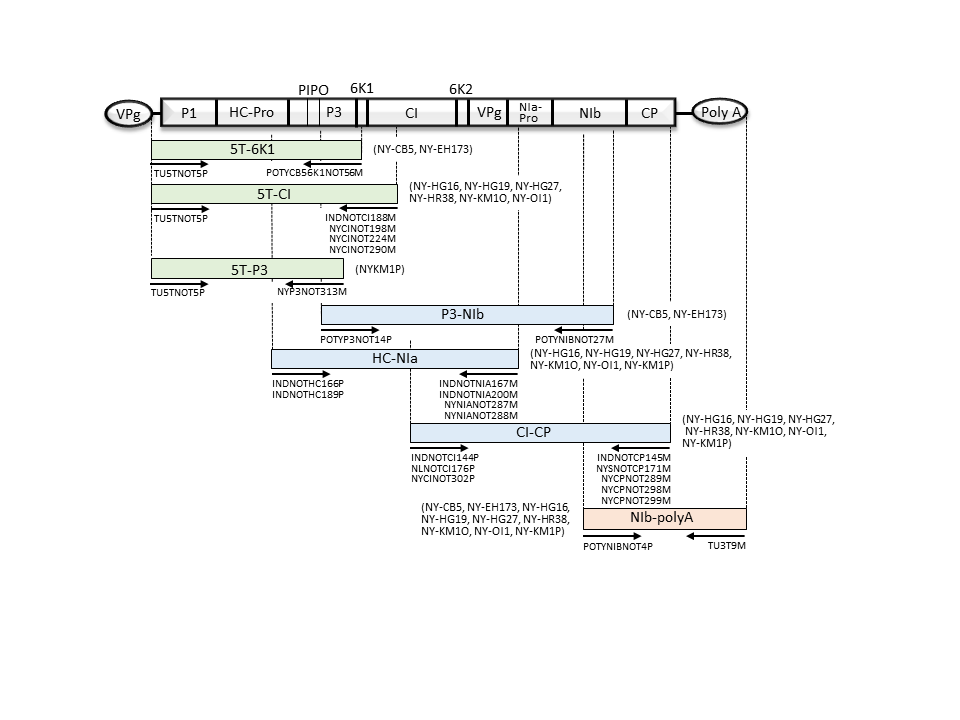

Supplement: S1 Fig — Arrow indicates primers used to amplify cDNA. Isolates are shown in parenthesis. The primer sequences are listed in S2 Table. (TIF) [file pone.0190511.s001.tif]

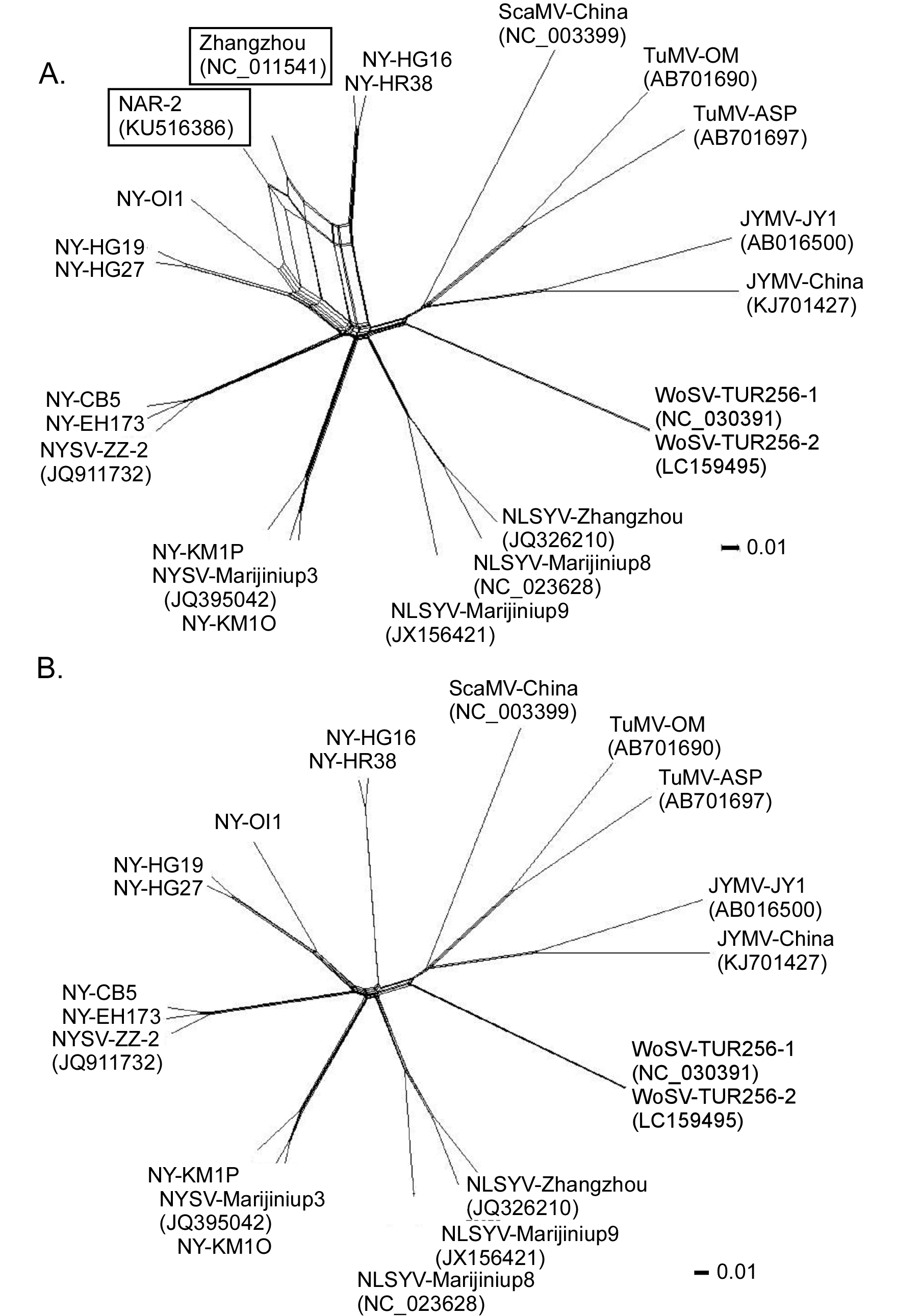

Supplement: S2 Fig — The sequences of polyproteins of narcissus yellow stripe virus (NYSV)-like viruses obtained in this study with those of outgroup sequences of Japanese yam mosaic virus (JYMV), narcissus late season yellows virus (NLSYV), scallion mosaic virus (ScaMV), turnip mosaic virus (TuMV) and wild onion symptomless virus (WoSV). Isolates with accession numbers were obtained from the public nucleotide sequence databases. Isolates NYSV Zhangzhou (NC_011541) and NYSV NAR-2 (KU516386) were added (A) or removed (B), and the trees were constructed. (TIF) [file pone.0190511.s002.tif]

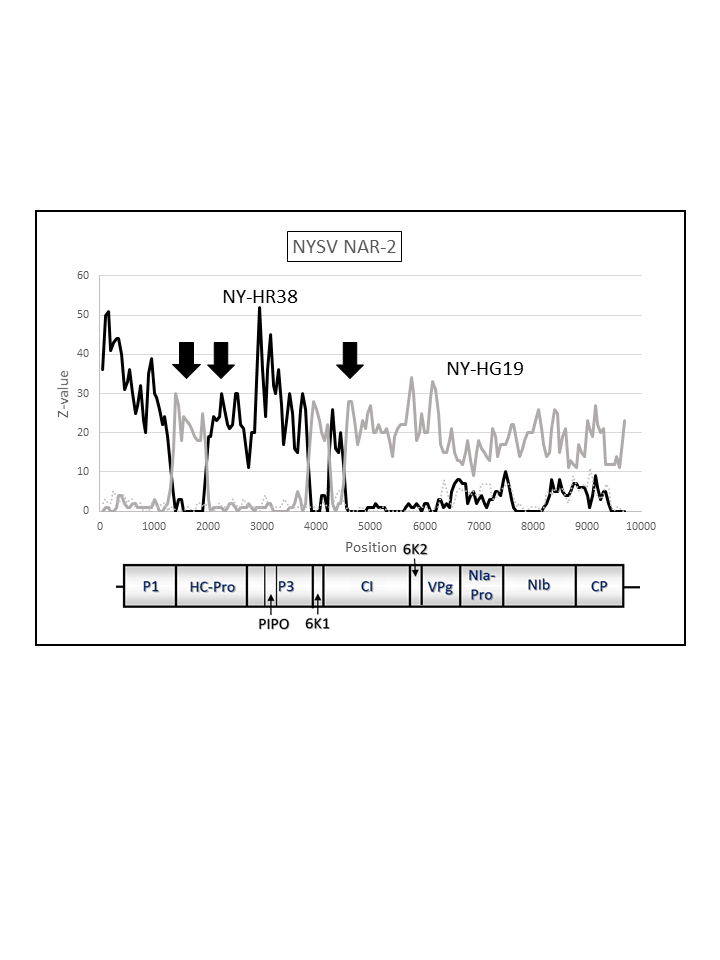

Supplement: S3 Fig — The sequences of NY-HR38 and NY-HG19 isolates represent the likely parental sequences of NYSV NAR-2 isolate. Note the support (i.e. z-value >3.0) for NYSV NAR-2 isolate being more closely related to NY-HR38 than NY-HG19 isolates in total nucleotide site analysis (see Table 2). The nucleotide positions are shown relative to the end of the degapped genome using the numbering of NAR-2 isolate. Arrows indicate the recombination sites of isolate NAR-2 identified using NY-HR38 and NY-HG19 sequences. For the graph, each window comparison involved sub-sequences of 100 nucleotides with a 50 nucleotides step between window positions. (TIF) [file pone.0190511.s003.tif]

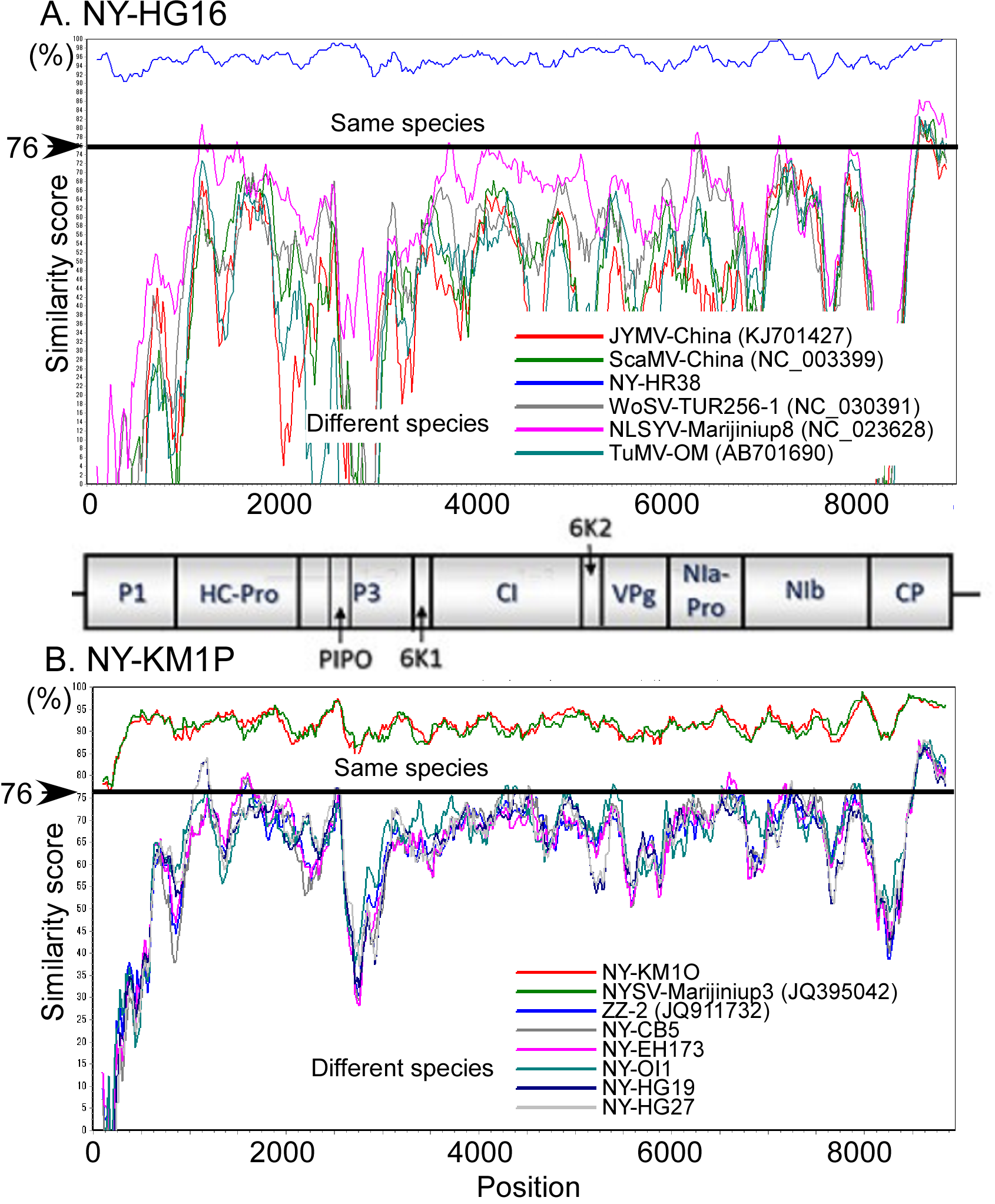

Supplement: S4 Fig — Isolates NY-HG16 (A) and NY-KM1P (B) were used as the query isolate. The similarities were estimated using SIMPLOT version 3.5.1 with a window size of 200 nt. (TIF) [file pone.0190511.s004.tif]

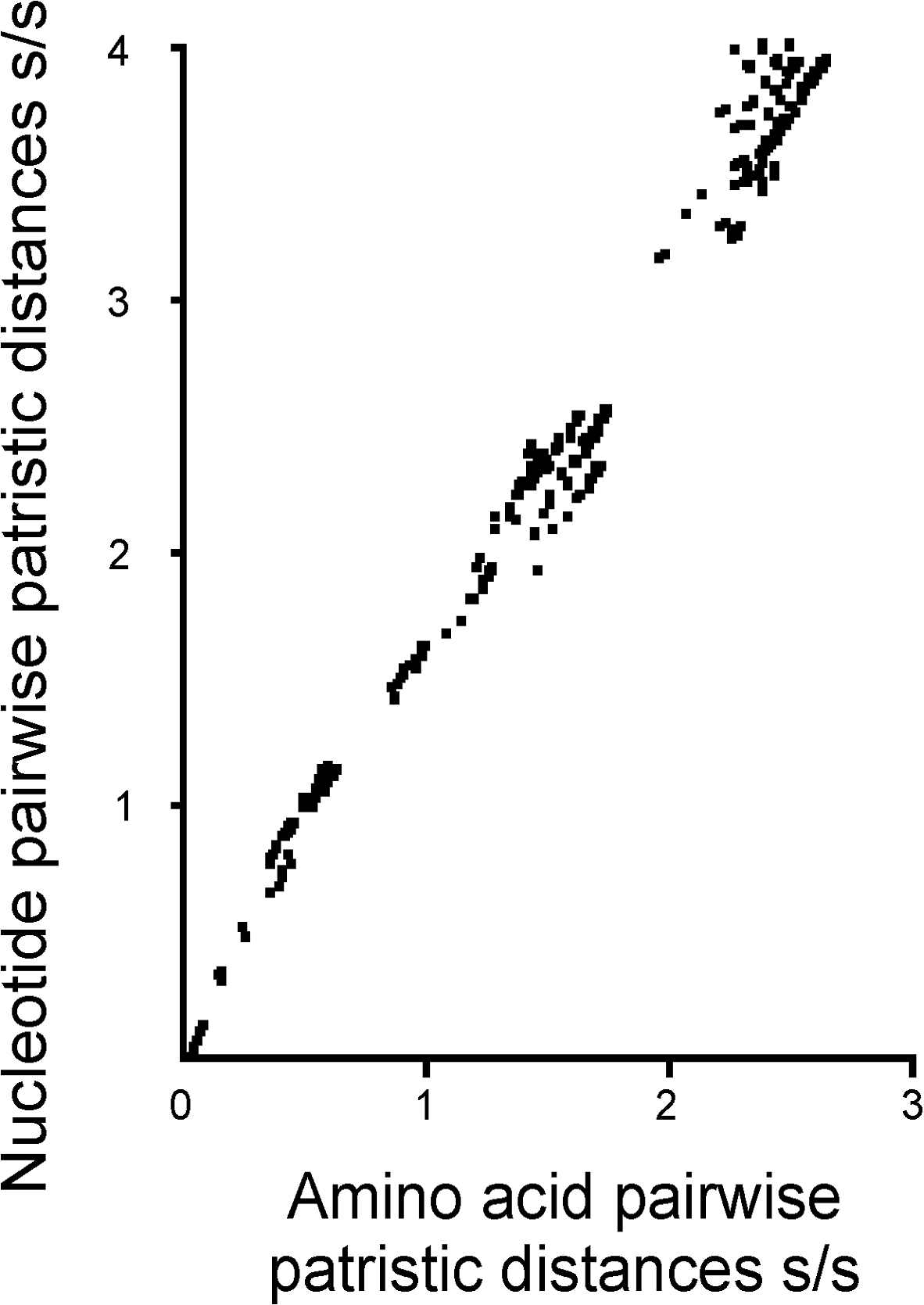

Supplement: S5 Fig — (TIF) [file pone.0190511.s005.tif]
