# Supplementary material for: The genetic diversity of narcissus viruses related to turnip mosaic virus blur arbitrary boundaries used to discriminate potyvirus species
Source: PLoS One. 2018 Jan 4;13(1):e0190511. doi: 10.1371/journal.pone.0190511 (PMC5754079; doi:10.1371/journal.pone.0190511)
Supplement: S1 Table — a Rows in brown show that Narcissus plants were infected with narcissus yellow stripe virus (NYSV)-like virus, whereas rows in blue show that those were infected with narcissus late season yellows virus (NLSYV), cyrtanthus elatus virus A (CyEVA), narcissus latent virus (NLV), narcissus degeneration virus (NDV) or ornithogalum mosaic virus (OrMV). b Number of clone sequenced for approximately 600–700 bp by POTYNIB5P primer. c Not detected. d Number of clone for NYSV-like virus sequence. (PDF) [file pone.0190511.s006.pdf]

S1 Table. Collection sites and the results of genetic diagnosis of *Narcissus* plants in this study.

Plant (Isolate)[a]	Location (town, city, prefecture)	Collection date	No. clone sequenced by POTYNIB5P	Detected virus
	Hokkaido district			
NY-HO1	Soen, Chuo-ku, Sapporo, Hokkaido	20 June 2012	7[b]	NLSYV
NY-HO2	Soen, Chuo-ku, Sapporo, Hokkaido	20 June 2012		ND[c]
NY-HO3	Tonden, Kita-ku, Sapporo, Hokkaido	21 June 2012		ND
NY-HO4	Tonden, Kita-ku, Sapporo, Hokkaido	21 June 2012		ND
NY-HO5	Tonden, Kita-ku, Sapporo, Hokkaido	24 June 2012		ND
NY-HO42	Nango, Shiroishi-ku, Sapporo, Hokkaido	1 June 2013	9	NLSYV
NY-HO43	Nango, Shiroishi-ku, Sapporo, Hokkaido	1 June 2013	8	NLSYV
NY-HO44	Kita 48 jo, Kita-ku, Sapporo, Hokkaido	2 June 2013	9	NLSYV
NY-HO45	Fujino, Minami-ku, Sapporo, Hokkaido	2 June 2013		ND
NY-HO46	Fujino, Minami-ku, Sapporo, Hokkaido	2 June 2013		ND
NY-HO47	Kitanosawa, Minami-ku, Sapporo, Hokkaido	2 June 2013		ND
NY-HO48	Maeda, Teine-ku, Sapporo, Hokkaido	2 June 2013	8	CyEVA
NY-HO49	Tonden, Kita-ku, Sapporo, Hokkaido	3 June 2013		ND
NY-HO50	Tonden, Kita-ku, Sapporo, Hokkaido	3 June 2013		ND
NY-HO51	Tonden, Kita-ku, Sapporo, Hokkaido	3 June 2013		ND
NY-HO52	Tonden, Kita-ku, Sapporo, Hokkaido	3 June 2013	10	NLSYV
NY-HO54	Minaminosato, Kitahirosima, Hokkaido	3 June 2013		ND
NY-HO55	Higashikyouei, Kitahirosima, Hokkaido	3 June 2013		ND
NY-HO56	Shinano, Chitose, Hokkaido	3 June 2013		ND
NY-HO186	Shinonome-cho, Chitose, Hokkaido	4 June 2014	9	NLV
NY-HO301	Maruyama, Kuriyama-cho, Yubari-gun, Hokkaido	6 May 2015	9	NLV
NY-HO306	Honcho, Naname-cho, Kameda-gun, Hokkaido	6 May 2015		ND
NY-HO307	Funamicho, Toyoura-cho, Abuta-gun, Hokkaido	7 May 2015	10	NLSYV
NY-HO308	Fukushima, KImobetsu-cho, Abuta-gun, Hokkaido	7 May 2015		ND
	Tohoku district			
NY-A65	Nambu-machi, Sannohe-gun, Aomori	22 January 2014	10	NLSYV
NY-A178	Nambu-machi, Sannohe-gun,Aomori	15 May 2014	11	NLSYV
NY-AK205	Sugisawaaratokoro, Yuzawa, Akita	14 June 2014		ND
NY-AK285	Hiroomote, Hiroomote, Akita Akita	25 April 2015		ND
NY-AK287	Nishinagano, Kadonodate-machi, Senboku, Akita	25 April 2015		ND
NY-AK288	Sotsuda, Kitazawako, Senboku, Akita	25 April 2015		ND
NY-AK289	Kyowahunooka, Daisen, Akita	25 April 2015		ND
NY-FK266	Motomiya, Fukushima	23 April 2015	9 (5[d])	NYSV-like, NLSYV
NY-FK270	Akogashima, Atami-machi, Koriyama, Fukushima	23 April 2015		ND
NY-FK275	Yamagata, Inawashiro-machi, Fukushima, Fukushima	23 April 2015		ND
NY-IW1	-, Morioka, Iwate	14 June 2014		ND
NY-IW2	-, Morioka, Iwate	14 June 2014		ND
NY-IW148	Toyama, Shiwa-cho, Shiwa-gun, Iwate	14 June 2014	10 (10)	NYSV-like
NY-IW149	Toyama, Shiwa-cho, Shiwa-gun, Iwate	14 June 2014	10 (10)	NYSV-like
NY-IW196	Koromogawa-ku, Oshu, Iwate	14 June 2014		ND
NY-IW203	Kawashiri, Nishiwaga-machi, Waga-gun, Iwate	14 June 2014		ND
NY-IW204	Kamiezuriko, Kitakami, Iwate	14 June 2014		ND
NY-MY210	Irimada, Shibata-machi, Shibata-gun, Miyagi	14 June 2014		ND
NY-MY211	Minamihase, Iwanuma, Miyagi	14 June 2014		ND
NY-MY292	Zao-machi, Katta, Miyagi	23 April 2015	8	NLSYV
NY-MY293	Zao-machi, Katta, Miyagi	23 April 2015	10 (5)	NYSV-like, NLSYV, NLV
NY-YA193	Zaohotta, Yamagata, Yamagata	15 June 2014		ND
NY-YA194	Hara-machi, Tendo, Yamagata	15 June 2014		ND
NY-YA195	Kubota-machi, Yonezawa, Yamagata	15 June 2014		ND
NY-YA276	-, Tsuruoka, Yamagata	25 April 2015		ND
	Kanto district			
NY-CB1	Tokashiro, Kimitsu, Chiba	26 March 2010	8 (1)	NYSV-like, NLSYV
NY-CB2	Sotominowa, Kimitsu, Chiba	26 March 2010	10 (8)	NYSV-like, NLSYV
NY-CB3	Sotominowa, Kimitsu, Chiba	26 March 2010	10 (1)	NYSV-like, NLSYV
NY-CB4	Ezuki, Kyonan-machi, Awa-gun, Chiba	26 March 2010	10	NLSYV
NY-CB5	Ezuki, Kyonan-machi, Awa-gun, Chiba	26 March 2010	10 (1)	NYSV-like, NLSYV, NDV
NY-CB9	Ezuki, Kyonan-machi, Awa-gun, Chiba	26 March 2010	9 (5)	NYSV-like, NLSYV
NY-CB91	Ezuki, Kyonan-machi, Awa-gun, Chiba	22 November 2013	8	NLSYV, NDV
NY-CB247	Ezuki, Kyonan-machi, Awa-gun, Chiba	17 March 2015	10 (6)	NYSV-like, NLSYV
NY-GM252	Fujiki, Tomioka, Gunma	17 March 2015	8	NLSYV
NY-GM253	Fujiki, Tomioka, Gunma	17 March 2015		ND
NY-GM254	Shiroishi, Fujioka, Gunma	17 March 2015		ND
NY-IB71A	Unknown, Ibaraki	11 November 2013	10	NLSYV
NY-IB248	Kanaya-cho, Mito, Ibaraki	17 March 2015	9	CyEVA
NY-KN1	Hasse-machi, Miura, Kanagawa	27 March 2010	10	NLSYV

[a] Rows in brown show that *Narcissus* plants were infected with narcissus yellow stripe virus (NYSV)-like virus, whereas rows in blue show that those were infected with narcissus late season yellows virus (NLSYV), cyrtanthus elatus virus A (CyEVA), narcissus latent virus (NLV), narcissus degeneration virus (NDV) or ornithogalum mosaic virus (OrMV).
[b] Number of clone sequenced for approximately 600-700 bp by POTYNIB5P primer
[c] Not detected
[d] Number of clone for NYSV-like virus sequence

S1 Table. Continued.

Plant (Isolate)	Location (town, city, prefecture)	Collection date	No. clones sequenced by POTYNIB5P	Detected virus
NY-KN2	Jogashima, Misaki, Miura, Kanagawa	27 March 2010		ND
NY-KN3	Jogashima, Misaki, Miura, Kanagawa	27 March 2010		ND
NY-KN4	Jogashima, Misaki, Miura, Kanagawa	27 March 2010		ND
NY-KN5	Jogashima, Misaki, Miura, Kanagawa	27 March 2010		ND
NY-KN6	Oba, Fujisawa, Kanagawa	27 March 2010	9 (1)	NYSV-like, NLSYV
NY-KN7	Oba, Fujisawa, Kanagawa	27 March 2010	9	NLSYV
NY-KN8	Sekiguchi, Atsugi, Kanagawa	28 March 2010	8	NLSYV
NY-KN9	-, Atsugi, Kanagawa	28 March 2010	9	OrMV
NY-ST1	Kamifukuoka, Fujimino, Saitama	28 March 2010	10 (10)	NYSV-like
NY-ST2	-, Toda, Saitama	30 March 2010	7 (2)	NYSV-like, NLSYV
NY-TC249	Ota, Fujioka-machi, Tochigi, Tochigi	17 March 2015		ND
NY-TC250	Osawa, Mashiko-machi, Haga-gun, Tochigi	17 March 2015	10	CyEVA
NY-TC251	Nogoya-machi, Utsunomiya, Tochigi	17 March 2015	10	NLSYV, NDV
NY-TK2	-, Koutou-ku, Tokyo	25 February 2010	7	NLSYV
NY-TK4	-, Akishima, Tokyo	28 March 2010	10 (5)	NYSV-like, NLSYV
NY-TK243	-, Ota-ku, Tokyo	17 March 2015	10	NLSYV
	Chubu district			
NY-AC230	Sannomaru, Naka-ku, Nagoya, Aichi	25 January 2015	11 (4)	NYSV-like, NLSYV
NY-AC233	Zoshi, Toyokawa, Aichi	25 January 2015	9	NLSYV, NDV
NY-AC234	Nagasawa-cho, Toyokawa, Aichi	25 January 2015		ND
NY-FI3	Hamakitayama, Fukui, Fukui	17 January 2012	8	NLSYV, NDV
NY-FI9	Hamakitayama, Fukui, Fukui	17 January 2012	10 (3)	NYSV-like, NLSYV
NY-FI11	Hamakitayama, Fukui, Fukui	17 January 2012	10	NLSYV, NDV
NY-FI15	Gamo, Fukui, Fukui	8 March 2012		ND
NY-FI23	Hamakitayama, Fukui, Fukui	8 March 2012	9	NDV
NY-FI29	Nashigadaira, Echizen-cho, Nyuu, Fukui	8 March 2012	10	NLSYV
NY-FI30	Nashigadaira, Echizen-cho, Nyuu, Fukui	8 March 2012	9	NLSYV, NDV
NY-FI33	Chigadaira, Echizen-cho, Nyuu, Fukui	8 March 2012	10	NLSYV
NY-NI7	-, -, Niigata	5 April 2010	5 (2)	NYSV-like
NY-NI260	Osato, Agano, Niigata	23 April 2015		ND
NY-NI263	Kuraoka, Konan-ku, Niigata, Niigata	24 April 2015	9	NLSYV
NY-NN75A	Hananoyamato, Daiichi Engei, Nagano	11 November 2013		ND
NY-SH216	Suzaki, Shimoda, Shizuoka	7 December 2014	10 (10)	NYSV-like
NY-SH219	Komakado, Gotemba, Shizuoka	7 December 2014	10	NLSYV
NY-SH236	Takasaki, Yaizu, Shizuoka	25 January 2015	9	NLSYV
NY-TY73A	Hananoyamato, Daiichi Engei, Toyama	11 November 2013	4	NLSYV
	Kinki district			
NY-HG1	-, Awaji, Hyogo	12 March 2011		ND
NY-HG3	Ichinomiya, Awaji, Hyogo	12 March 2011	8	NYSV-like, CyEVA
NY-HG5	Ichinomiya, Awaji, Hyogo	12 March 2011		ND
NY-HG6	Ichinomiya, Awaji, Hyogo	12 March 2011		ND
NY-HG8	Nadakuroiwa, Minamiawaji, Hyogo	12 March 2011	9 (5)	NYSV-like, NLSYV, NDV
NY-HG10	Nadakuroiwa, Minamiawaji, Hyogo	12 March 2011	10 (1)	NYSV-like, NLSYV, NDV
NY-HG11	ura-cho, Sumoto, Hyogo	12 March 2011	10 (10)	NYSV-like
NY-HG13	Hon-machi, Himeji, Hyogo	14 March 2011		ND
NY-HG16	Amida-cho, Takasago, Hyogo	14 March 2011	6 (5)	NYSV-like, NLSYV
NY-HG19	Kukuchinishi-cho, Amagasaki, Hyogo	10 March 2012	10 (10)	NYSV-like
NY-HG20	Meishin, Amagasaki, Hyogo	10 March 2012	9	NLSYV, NDV
NY-HG25	Akurakita, Takarazuka, Hyogo	10 March 2012	10	NLSYV
NY-HG24	Sakae, Takarazuka, Hyogo	10 March 2012		ND
NY-HG27	Kamo, Kawanishi, Hyogo	10 March 2012	7 (7)	NYSV-like
NY-KY83	-, -, Kyoto	19 November 2013		ND
NY-KY84	-, -, Kyoto	19 November 2013		ND
NY-ME224	Koazaka-cho, Matsuzaka, Mie	24 January 2015	9	NLSYV
NY-ME226	Shihikida, Taki-cho, Taki-gun, Mie	24 January 2015		ND
NY-ME229	Komei-cho, Tsu, Mie	24 January 2015	9 (2)	NYSV-like, NDV
NY-OS1	Minowa, Toyonaka, Osaka	10 March 2012	8 (3)	NYSV-like, NLSYV
NY-OS2	Himemuro, Ikeda, Osaka	10 March 2012	7	NLSYV
	Chugoku district			
NY-HR36	Yanohigashi, Akiku, Hiroshima, Hiroshima	8 March 2013	7 (7)	NYSV-like
NY-HR38	Ushirogake, Kawajiri, Kure, Hiroshima	8 March 2013	9 (9)	NYSV-like
NY-HR39	Sunami, Mihara, Hiroshima	8 March 2013	7	NLSYV
NY-HR40	Higashiteshiro, Fukuyama, Hiroshima	9 March 2013	6	NDV
NY-OY1	Kotsu, Higashi-ku, Okayama, Okayama	13 March 2011	9 (5)	NYSV-like, NLSYV
NY-OY41	Kuma, Kita-ku, Okayama, Okayama	9 March 2013		ND
NY-SM69A	Unknown, Shimane	11 November 2013	10	NLSYV, OrMV
NY-YM2	Chinto, Hagi, Yamaguchi	26 February 2011	10	NLSYV, NDV
NY-YM5	Nabe-cho, Shimonoseki, Yamaguchi	27 February 2011	10 (2)	NYSV-like, NLSYV, NDV
	Shikoku district			
NY-EH171	Takanoko-cho, Matsuyama, Ehime	26 April 2014	10	NYSV-like, NDV
NY-EH173	Kurumi, Tambara-cho, Saijo, Ehime	26 April 2014	10	NYSV-like
NY-KO163	Otesuji, Kochi, Kochi	25 April 2014		ND
NY-KO164	Aioi-cho, Kochi, Kochi	25 April 2014	9 (9)	NYSV-like
NY-KO165	Ikku, Kochi, Kochi	25 April 2014		ND
NY-KW1	Ejiri-cho, Sakaide, Kagawa	13 March 2011	6	NLSYV

S1 Table. Continued.

Plant (Isolate)	Location (town, city, prefecture)	Collection date	No. clones sequenced by POTYNIB5P	Detected virus
NY-KW2	Oumi, Sakaide, Kagawa	13 March 2011	3	CyEVA
NY-KW3	Oumi, Sakaide, Kagawa	13 March 2011	10 (10)	NYSV-like
NY-KW4	Ikushima-cho, Takamatsu, Kagawa	13 March 2011	8	NLSYV
NY-KW5	Nakayama-cho, Takamatsu, Kagawa	13 March 2011	7	OrMV
NY-KW6	Danshi-cho, Takamatsu, Kagawa	13 March 2011	8 (8)	NYSV-like
NY-TS158	Kizuno, Otsu-cho, Naruto, Tokushima	24 April 2014		NLV
NY-TS159	Kizuno, Otsu-cho, Naruto, Tokushima	24 April 2014		ND
NY-TS160	Yoshinaga, Otsu-cho, Naruto, Tokushima	26 April 2014		ND
NY-TS161	-, Mima, Tokushima	26 April 2014		ND
	Kyushu district			
NY-F1	-, Okawa, Fukuoka	20 March 2010	10 (3)	NYSV-like, NLSYV, NDV
NY-F2	Yamakawa-machi, Miyama, Fukuoka	24 January 2011	7	NLSYV
NY-F8	Kawabaru, Itoshima, Fukuoka	11 December 2011	9	NLSYV
NY-F9	Misaka, Itoshima, Fukuoka	11 December 2011	6 (6)	NYSV-like
NY-KG1	Tokunoshima-cho, Oshima, Kagoshima	22 January 2010	10 (10)	NYSV-like
NY-KG2	Tokunoshima-cho, Oshima, Kagoshima	22 January 2010	9 (3)	NYSV-like, NLSYV
NY-KG6	Tenokuchi, Kagoshima, Kagoshima	16 February 2012	9	NLSYV
NY-KG7	Tenokuchi, Kagoshima, Kagoshima	16 February 2012		ND
NY-KG8	Yamanokuchi, Kagoshima, Kagoshima	16 February 2012		ND
NY-KG9	Uenosono, Kagoshima, Kagoshima	16 February 2012	6 (2)	NYSV-like, NLSYV
NY-KG10	Uearata-cho, Kagoshima, Kagoshima	16 February 2012	6 (1)	NYSV-like, NDV
NY-KG11	Uearata-cho, Kagoshima, Kagoshima	16 February 2012	6	NDV
NY-KG12	Arata-cho, Kagoshima, Kagoshima	16 February 2012	9 (9)	NYSV-like
NY-KG13	Uearata-cho, Kagoshima, Kagoshima	16 February 2012	10	NLSYV
NY-KM1O	Miyabara, Arao, Kumamoto	23 January 2011	10	NYSV-like
NY-KM1P	Miyabara, Arao, Kumamoto	23 January 2011	10	NYSV-like
NY-KM9	Ozu-machi, Kikuchi, Kumamoto	28 March 2011	6	NLSYV, NDV
NY-M1	Higashinagaeura, Ebino, Miyazaki	7 May 2012	3	NLV
NY-M2	Higashinagaeura, Ebino, Miyazaki	7 May 2012	7 (6)	NYSV-like, CyEVA
NY-M3	Suenaga, Ebino, Miyazaki	7 May 2012		ND
NY-M4	Takaharu, Nishimorokata-gun, Miyazaki	7 May 2012	10	NLSYV
NY-M5	Takazaki-cho, Miyakonojo, Miyazaki	7 May 2012		ND
NY-M77	-, -, Miyazaki	15 November 2013		ND
NY-N2	Kunimi-cho, Unzen, Nagasaki	6 March 2009		ND
NY-N6	Kayaki-cho, Nagasaki, Nagasaki	27 February 2010		ND
NY-N9	Kurohama-machi, Nagasaki, Nagasaki	27 February 2010	8	NLSYV
NY-N12	Nomozaki, Nomo-cho, Nagasaki, Nagasaki	27 February 2010	8	NLSYV
NY-N13	Nomozaki, Nomo-cho, Nagasaki, Nagasaki	27 February 2010		ND
NY-N14	Nomozaki, Nomo-cho, Nagasaki, Nagasaki	27 February 2010	9 (7)	NYSV-like, NLSYV, NDV
NY-N15	Uki-machi, Isahaya, Nagasaki	27 February 2010	10 (6)	NYSV-like, NLSYV
NY-N18	Obama-cho, Unzen, Nagasaki	28 February 2010		ND
NY-N19	Arima-cho, Minamishimabara, Nagasaki	28 February 2010	15 (1)	NYSV-like, NLSYV, CyEVA
NY-N20	Ariake-cho, Shimabara, Nagasaki	28 February 2010	10 (10)	NYSV-like
NY-OI1	Takasakiyama, Kozaki, Oita, Oita	26 February 2012	10 (10)	NYSV-like
NY-OI2	Takasakiyama, Kozaki, Oita, Oita	26 February 2012	10 (10)	NYSV-like
NY-OI3	Takasakiyama, Kozaki, Oita, Oita	26 February 2012	10 (10)	NYSV-like
NY-OI4	Tanoyu-machi, Beppu, Oita	26 February 2012	8	NLSYV
NY-OI5	Tanoyu-machi, Beppu, Oita	26 February 2012	8	NYSV-like
NY-OI12	Fujimi-cho, Beppu, Oita	26 February 2012	9 (6)	NYSV-like, NLSYV
NY-OI13	Tenman-cho, Beppu, Oita	26 February 2012	10 (10)	NYSV-like
NY-OI14	Tenman-cho, Beppu, Oita	26 February 2012		ND
NY-SG8	Hyogo-machi, Saga, Saga	14 March 2010	10	NLSYV
NY-SG10	Honjo-machi, Saga, Saga	14 January 2011		ND
NY-SG12	Honjo-machi, Saga, Saga	8 January 2013	9	NLSYV, NDV
	Okinawa district			
NY-OK98	Kyoda, Nago, Okinawa	25 January 2014	10 (10)	NYSV-like
NY-OK100	Kyoda, Nago, Okinawa	25 January 2014	6 (6)	NYSV-like
