# Supplementary material for: The genetic diversity of narcissus viruses related to turnip mosaic virus blur arbitrary boundaries used to discriminate potyvirus species
Source: PLoS One. 2018 Jan 4;13(1):e0190511. doi: 10.1371/journal.pone.0190511 (PMC5754079; doi:10.1371/journal.pone.0190511)
Supplement: S2 Table — a Correspond to the genome of Chinese isolate [1]. b P1; Protein 1, HC-Pro; Helper component-proteinase protein, P3; Protein 3, 6K1; 6Kda 1 protein, CI; Cylindrical inclusion protein, 6K2; 6Kda 2 protein, VPg; Genome-linked viral protein, NIa-Pro; Nuclear inclusion a-proteinase protein, NIb; Nuclear inclusion b protein, CP; Coat protein. c R; G+A, Y; C+T, S; G+C, M; A+C, W; A+T, K, T+G, V; A+C+G, D; A+T+G, H; A+T+C, N; A+T+G+C, GCGGCCGC; NotI restriction site. d Underlined sequence is the position of POTYNIB5P primer (5’- CGCATATGGGGTGAGAGAGG- 3’) used for sequencing. (PDF) [file pone.0190511.s007.pdf]

S2 Table. Primers used to amplify RT-PCR products in this study.

Primer name	Sense	Position[a]	Region/Gene[b]	Sequence (5'-3')[c]
TU5TNOT5P	Plus	1-24	5'NCR	GGGGCGGCCGCAAAAAATATAAAAACTCAACACAACA
INDNOTHC189P	Plus	2133-2155	HC-Pro	GGGGCGGCCGCTCTTCTATCCTGATATTAGCAGTG
INDNOTHC166P	Plus	2162-2184	HC-Pro	GGGGCGGCCGCGCTAAAGCATTCACDAAGATGGT
POTYP3NOT14P	Plus	3072-3093	P3	GGGGCGGCCGCATATGGGGTGAGAGAGGGATTTAGGMGGCAGATACAGCGA
NYP3NOT313M	Minus	3155-3174	P3	GGGGCGGCCGCTAACTAGCTAGTTTGTCCCTA
POTYCB56K1NOT56M	Minus	3527-3549	6K1	GGGGCGGCCGCACGCGCTCTAATTCTCGTTCTGA
INDNOTCI188M	Minus	3840-3840	CI	GGGGCGGCCGCGCTCGTGTAAATGTAACTTGAAACC
NYCINOT198M	Minus	3902-3924	CI	GGGGCGGCCGCACAAGTAGAATGTCGTTTCGTTC
NYCINOT290M	Minus	3902-3923	CI	GGGGCGGCCGCTCAATAGAATGTCTCTCTTGTC
INDNOTCI144P	Plus	4661-4683	CI	GGGGCGGCCGCCTSGAYWGYGACAAYMGAATGAT
NLNOTCI176P	Plus	4970-4991	CI	GGGGCGGCCGCTTTGAYGGNTCRATGCAYCCAG
NYCINOT302P	Plus	4990-5011	CI	GGGGCGGCCGCTGCACTCCATGCCCTGTTCAAG
NYCINOT224M	Minus	5000-5020	CI	GGGGCGGCCGCCTTGAACCTCTTGAACAGGGC
INDNOTNIA167M	Minus	6522-6545	NIa-Pro	GGGGCGGCCGCGAGTAGTGTTGCGGATTAGGAACT
INDNYNIANOT200M	Minus	6527-6548	NIa-Pro	GGGGCGGCCGCATTGTGTTGTATTCCTGATTGA
NYNIANOT288M	Minus	6586-6606	NIa-Pro	GGGGCGGCCGCAACATCTTTCGGCAACCGCAAG
NYNIANOT287M	Minus	6640-6660	NIa-Pro	GGGGCGGCCGCATACGTTCATTTCCAATGGGC
POTYNIBNOT4P[d]	Plus	7667-7689	NIb	GGGGCGGC<u>CGCATATGGGGTGAGAGAGG</u>TNTGYGTNGAYGAYTTYAAYAA
POTYNIBNOT27M	Minus	8161-8182	NIb	GGGGCGGCCGCAAACCTGGCTGACAGTGTGTCA
NYCPNOT289M	Minus	8685-8706	CP	GGGGCGGCCGCTTCCGCACTTCACTGGCGACAC
NYCPNOT298M	Minus	8694-8715	CP	GGGGCGGCCGCGTCCCTACTTTACTCGGTTCCT
NYCPNOT299M	Minus	8809-8829	CP	GGGGCGGCCGCACAACTGGCTTACCACCACTC
NYSNOTCP171M	Minus	8810-8832	CP	GGGGCGGCCGCTTCACAACTGGCTTTCCACCACT
NYCPNOT286M	Minus	8820-8841	CP	GGGGCGGCCGCTGCCCCAAGTTTAAGATTGGAC
INDNOTCP145M	Minus	8836-8861	CP	GGGGCGGCCGCTTGGTGAATAAGATAACAAATGCTCT
TU3T9M	Minus	polyA	polyA	GGGGCGGCCGCT$_{15}$

[a] Correspond to the genome of Chinese isolate [1]

[b] P1; Protein 1, HC-Pro; Helper component-proteinase protein, P3; Protein 3, 6K1; 6Kda 1 protein, CI; Cylindrical inclusion protein, 6K2; 6Kda 2 protein, VPg; Genome-linked viral protein, NIa-Pro; Nuclear inclusion a-proteinase protein, NIb; Nuclear inclusion b protein, CP; Coat protein.

[c] R; G+A, Y; C+T, S; G+C, M; A+C, W; A+T, K, T+G, V; A+C+G, D; A+T+G, H; A+T+C, N; A+T+G+C, GCGGCCGC; NotI restriction site

[d] Underlined sequence is the position of POTYNIB5P primer (5'- CGCATATGGGGTGAGAGAGG- 3') used for sequencing.

Supporting references

1. Chen J, Lu YW, Shi YH, Adams MJ, Chen JP. Complete nucleotide sequence of the genomic RNA of narcissus yellow stripe virus from Chinese narcissus in Zhangzhou city, China. Arch Virol. 2006;151,1673–1677.
