# Supplementary material for: The genetic diversity of narcissus viruses related to turnip mosaic virus blur arbitrary boundaries used to discriminate potyvirus species
Source: PLoS One. 2018 Jan 4;13(1):e0190511. doi: 10.1371/journal.pone.0190511 (PMC5754079; doi:10.1371/journal.pone.0190511)
Supplement: S3 Table — a NYSV; narcissus yellow stripe virus, NLSYV; narcissus late season yellows virus. b P1; protein 1, HC-Pro; helper component-proteinase protein, P3; protein 3, 6K1; 6kda 1 protein, CI; cylindrical inclusion protein, 6K2; 6kda 2 protein, VPg; genome-linked viral protein, NIa-Pro; nuclear inclusion a-proteinase protein, NIb; nuclear inclusion b protein, CP; coat protein. c Numbers colored in red show different amino acid residues at cleavage sites. (PDF) [file pone.0190511.s008.pdf]

S3 Table. Tentative amino acid residues at the cleavage sites between protein coding regions.

Phylogenetic group and isolate[a]	Amino acid residues of cleavage site									Stop codon
	P1/HC-Pro[b]	HC-Pro/P3	P3/6K1	6K1/CI	CI/6K2	6K2/VPg	VPg/NIa-Pro	NIa-Pro/NIb	NIb/CP	
Narcissus virus-1 (NV-1)										
NY-HG16	Y/S[c]	G/G	Q/V	Q/S	Q/S	E/A	E/S	Q/M	Q/S	TAA
NY-HR38	Y/S	G/G	Q/T	Q/S	Q/S	E/A	E/S	Q/M	Q/S	TAA
NYSV-1										
NY-KM1O	Y/S	G/G	Q/T	Q/S	Q/S	E/A	E/S	Q/M	Q/S	TAA
NY-KM1P	Y/S	G/G	Q/T	Q/S	Q/S	E/A	E/S	Q/M	Q/S	TAA
Marijiniup3 (JQ395042)	Y/S	G/G	Q/T	Q/S	Q/S	E/A	E/S	Q/M	Q/S	TAA
NYSV-2										
NY-OI1	Y/A	G/G	Q/V	Q/S	Q/S	E/A	E/S	Q/M	Q/S	TAA
NY-HG19	Y/A	G/G	Q/A	Q/S	Q/S	E/A	E/S	Q/M	Q/S	TAA
NY-HG27	Y/A	G/G	Q/V	Q/S	Q/S	E/A	E/S	Q/M	Q/S	TAA
NYSV-3										
ZZ-2 (JQ911732)	Y/A	G/G	Q/A	Q/S	Q/S	E/A	E/S	Q/M	Q/S	TAG
NY-CB5	Y/A	G/G	Q/A	Q/S	Q/S	E/A	E/S	Q/M	Q/S	TAG
NY-EH173	Y/A	G/G	Q/A	Q/S	Q/S	E/A	E/S	Q/M	Q/S	TAG
Recombinant										
Zhangzhou (NC_011541)	Y/S	G/G	Q/T	Q/S	Q/S	E/A	E/S	Q/M	Q/S	TAA
NAR-2 (KU516386)	Y/S	G/G	Q/T	Q/S	Q/S	E/A	E/S	Q/M	Q/S	TAA
NLSYV										
Zhangzhou (JQ326210)	F/S	G/G	Q/V	Q/S	Q/S	E/A	E/S	Q/M	Q/S	TAA
Marijiniup8 (NC_023628)	F/T	G/G	Q/V	Q/S	Q/S	E/A	E/S	Q/M	Q/S	TAA
Marijiniup9 (JX156421)	F/T	G/G	Q/A	Q/S	Q/S	E/A	E/S	Q/M	Q/S	TAA

[a] NYSV; narcissus yellow stripe virus, NLSYV; narcissus late season yellows virus

[b] P1; protein 1, HC-Pro; helper component-proteinase protein, P3; protein 3, 6K1; 6kda 1 protein, CI; cylindrical inclusion protein, 6K2; 6kda 2 protein, VPg; genome-linked viral protein, NIa-Pro; nuclear inclusion a-proteinase protein, NIb; nuclear inclusion b protein, CP; coat protein

[c] Numbers colored in red show different amino acid residues at cleavage sites.
