# Supplementary material for: The genetic diversity of narcissus viruses related to turnip mosaic virus blur arbitrary boundaries used to discriminate potyvirus species
Source: PLoS One. 2018 Jan 4;13(1):e0190511. doi: 10.1371/journal.pone.0190511 (PMC5754079; doi:10.1371/journal.pone.0190511)
Supplement: S4 Table — Percent identities of protein 1 and cytoplasmic inclusion coding regions were calculated by EMBOSS Needle [2]. The identity values greater than the thresholds of species demarcation criteria are shown in red letter. Potyvirus species demarcation criteria of protein 1 and cytoplasmic inclusion coding regions are <58% and <76% nucleotide identities, respectively [3, 4]. (PDF) [file pone.0190511.s009.pdf]

S4 Table. Comparison of the percentage identical nucleotides in the protein 1 (bottom left) and cylindrical inclusion protein (top right) coding regions of narcissus yellow stripe virus-like virus and narcissus late season yellows virus isolates.

Virus and isolate	narcissus virus 1		narcissus late season yellows virus			narcissus yellow stripe virus-like virus								
						NYSV-1			NYSV-2			NYSV-3		
	NY-HG16	NY-HR38	Marijiniup8	Marijiniup9	Zhangzhou	Marijiniup3	NY-KM1O	NY-KM1P	NY-CB5	NY-EH173	ZZ-2	NY-HG19	NY-HG27	NY-OI1
NY-HG16	-	95.4	74.9	74.3	73.6	74.2	74.2	74.1	74.1	74.2	74.5	74.7	74.2	73.9
NY-HR38	93.1	-	75.1	74.2	73.6	74.7	74.5	74.2	74.2	74.4	74.4	74.5	74.3	74.3
Marijiniup8	52.8	49.7	-	82.0	81.9	75.2	75.3	75.1	74.4	74.0	73.7	75.9	75.3	75.3
Marijiniup9	54.4	52.0	76.8	-	89.8	74.3	74.5	74.9	73.7	73.9	73.5	74.3	74.8	73.5
Zhangzhou	54.8	53.4	77.1	90.2	-	74.4	75.0	75.0	73.4	73.9	73.3	73.9	74.4	74.1
Marijiniup3	55.8	56.0	52.6	54.0	55.0	-	95.7	91.6	75.7	75.2	75.4	74.2	75.1	75.7
NY-KM1O	56.4	55.6	52.3	52.9	53.8	96.0	-	92.3	75.7	74.8	75.2	76.1	75.5	76.0
NY-KM1P	55.3	53.1	54.5	53.4	55.8	88.3	88.1	-	76.2	75.0	75.3	76.2	75.6	76.3
NY-CB5	50.8	48.7	51.8	52.1	53.6	57.3	57.7	58.8	-	95.5	95.2	76.9	77.5	76.2
NY-EH173	51.8	50.7	54.6	52.0	52.3	56.9	56.6	58.1	94.1	-	95.1	76.8	77.2	75.6
ZZ-2	52.2	50.7	52.1	51.1	52.6	55.5	55.9	59.1	90.6	91.6	-	77.2	77.6	76.8
NY-HG19	54.0	52.0	50.0	54.6	53.6	56.0	56.8	56.4	58.4	57.3	57.7	-	95.9	82.9
NY-HG27	54.9	54.8	50.8	54.3	54.0	55.3	55.9	54.0	58.4	56.3	57.7	94.4	-	82.3
NY-OI1	55.2	54.5	52.6	56.1	56.8	55.4	57.0	58.1	55.7	56.9	58.3	69.0	67.4	-

Percent identities of protein 1 and cytoplasmic inclusion coding regions were calculated by EMBOSS Needle [2]. The identity values greater than the thresholds of species demarcation criteria are shown in red letter. Potyvirus species demarcation criteria of protein 1 and cytoplasmic inclusion coding regions are <58% and <76% nucleotide identities, respectively [3, 4].

Supporting references

2. Rice P, Longden I, Bleasby A. EMBOSS: The European Molecular Biology Open Software. Suite Trends Genet. 2000;16, 276–277.
3. Adams MJ, Antoniw JF, Fauquet CM. Molecular criteria for genus and species discrimination within the family Potyviridae. Arch Virol. 2005;150, 459–479.
4. Wylie SJ, Adams M, Chalam C, Kreuze J, López-Moya J, Ohshima K, Praveen S, Rabenstein F, Stenger D, Wang A, Zerbini FM, ICTV Consortium. ICTV Virus Taxonomy Profile: Potyviridae. J Gen Virol. 2017;98, 352-354
